# Supplementary material for: Origins of the 2009 H1N1 influenza pandemic in swine in Mexico
Source: eLife. 2016 Jun 28;5:e16777. doi: 10.7554/eLife.16777 (PMC4957980; doi:10.7554/eLife.16777)
Supplement: Figure 3—source data 1. — The color of each branch indicates the most probable location state, similar to Figure 3. Clades of Mexican viruses are labeled. Posterior probabilities >80 are provided for key nodes. The 95% HPD values for the estimated tMRCA also are provided for key nodes with light blue bars. Viruses with genotype 1 similar to pdmH1N1 are indicated with green stars on the PB2 tree; a more detailed presentation of the evolution of genotypes in Mexico in all trees is provided in Figure 3—source data 3. DOI: http://dx.doi.org/10.7554/eLife.16777.008 [file elife-16777-fig3-data1.pdf]

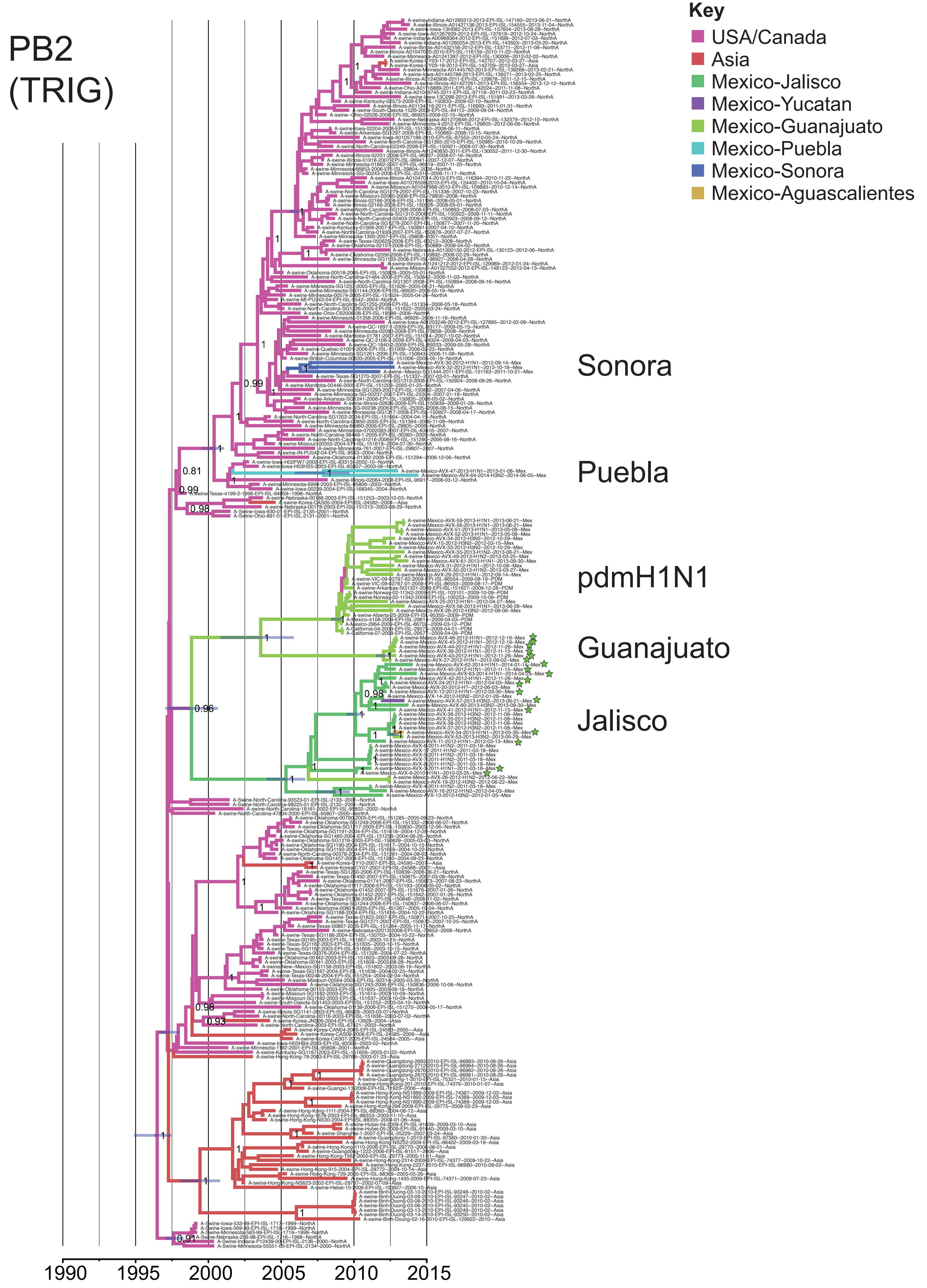
PB2
(TRIG)
Key
USA/Canada
Asia
Mexico-Jalisco
Mexico-Yucatan
Mexico-Guanajuato
Mexico-Puebla
Mexico-Sonora
Mexico-Aguascalientes
Sonora
Puebla
pdmH1N1
Guanajuato
Jalisco
1990
1995
2000
2005
2010
2015

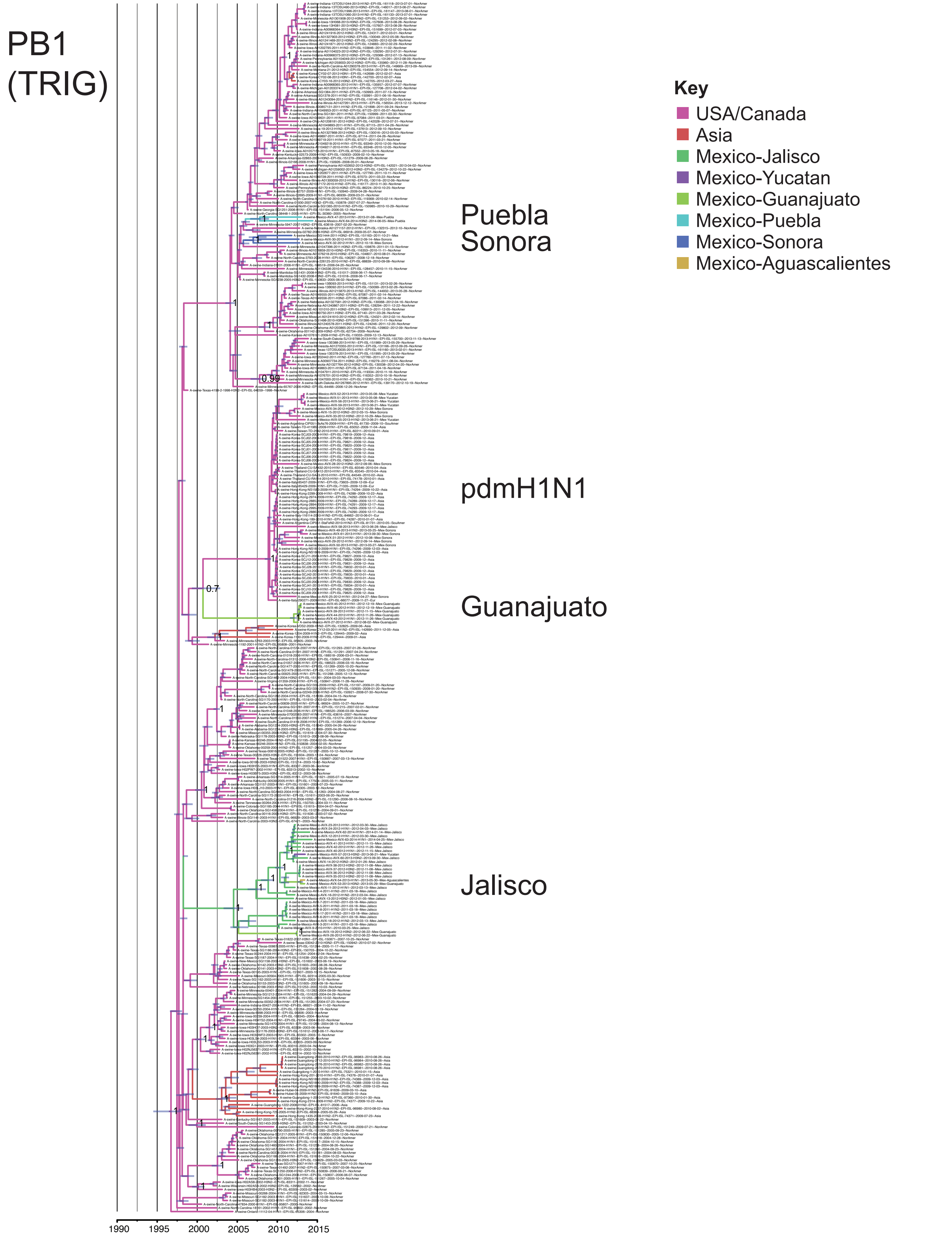

PB1
(TRIG)
Key
USA/Canada
Asia
Mexico-Jalisco
Mexico-Yucatan
Mexico-Guanajuato
Mexico-Puebla
Mexico-Sonora
Mexico-Aguascalientes
Puebla
Sonora
pdmH1N1
Guanajuato
Jalisco
1990
1995
2000
2005
2010
2015

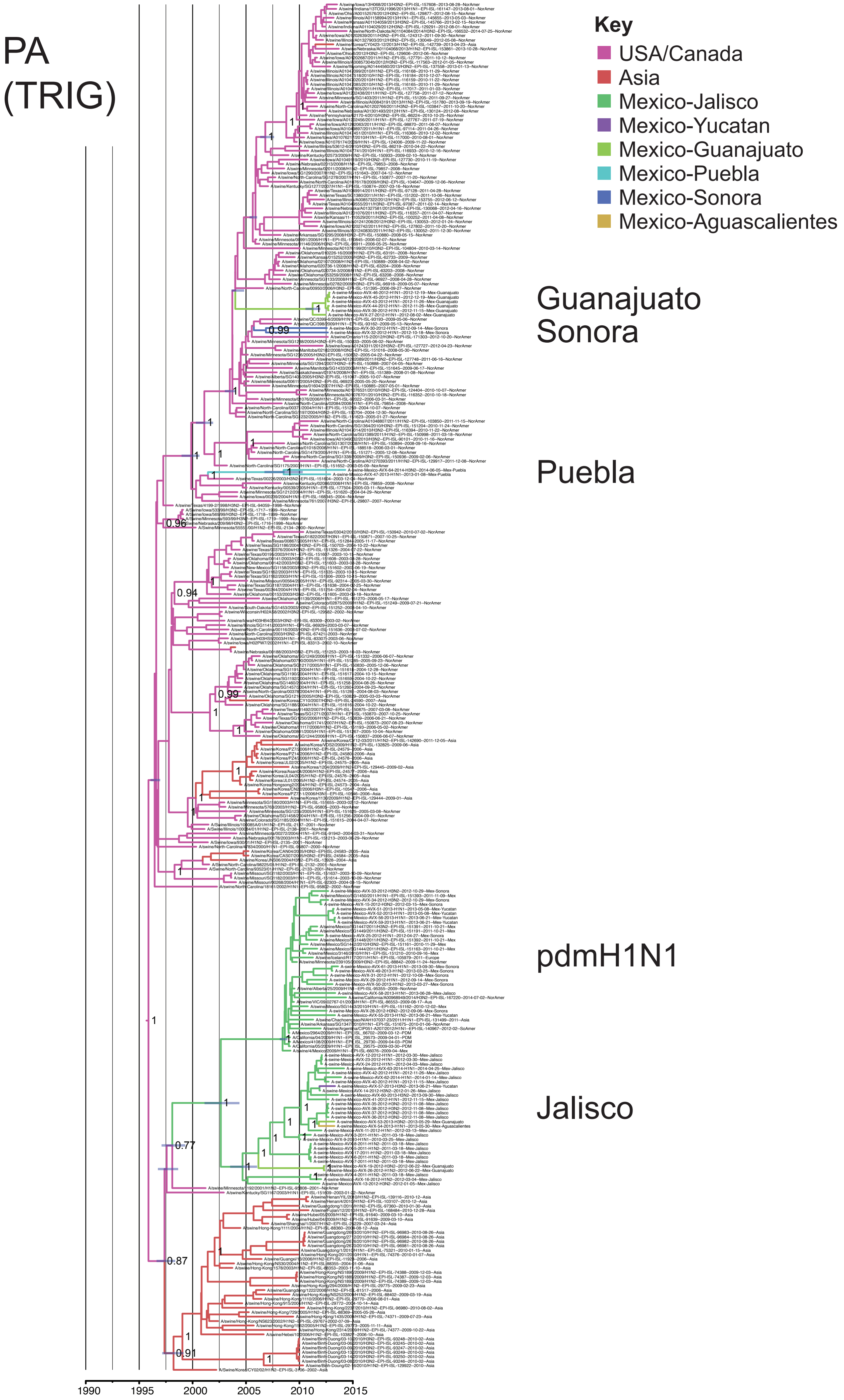
PA
(TRIG)
Key
USA/Canada
Asia
Mexico-Jalisco
Mexico-Yucatan
Mexico-Guanajuato
Mexico-Puebla
Mexico-Sonora
Mexico-Aguascalientes
Guanajuato
Sonora
Puebla
pdmH1N1
Jalisco
1990
1995
2000
2005
2010
2015

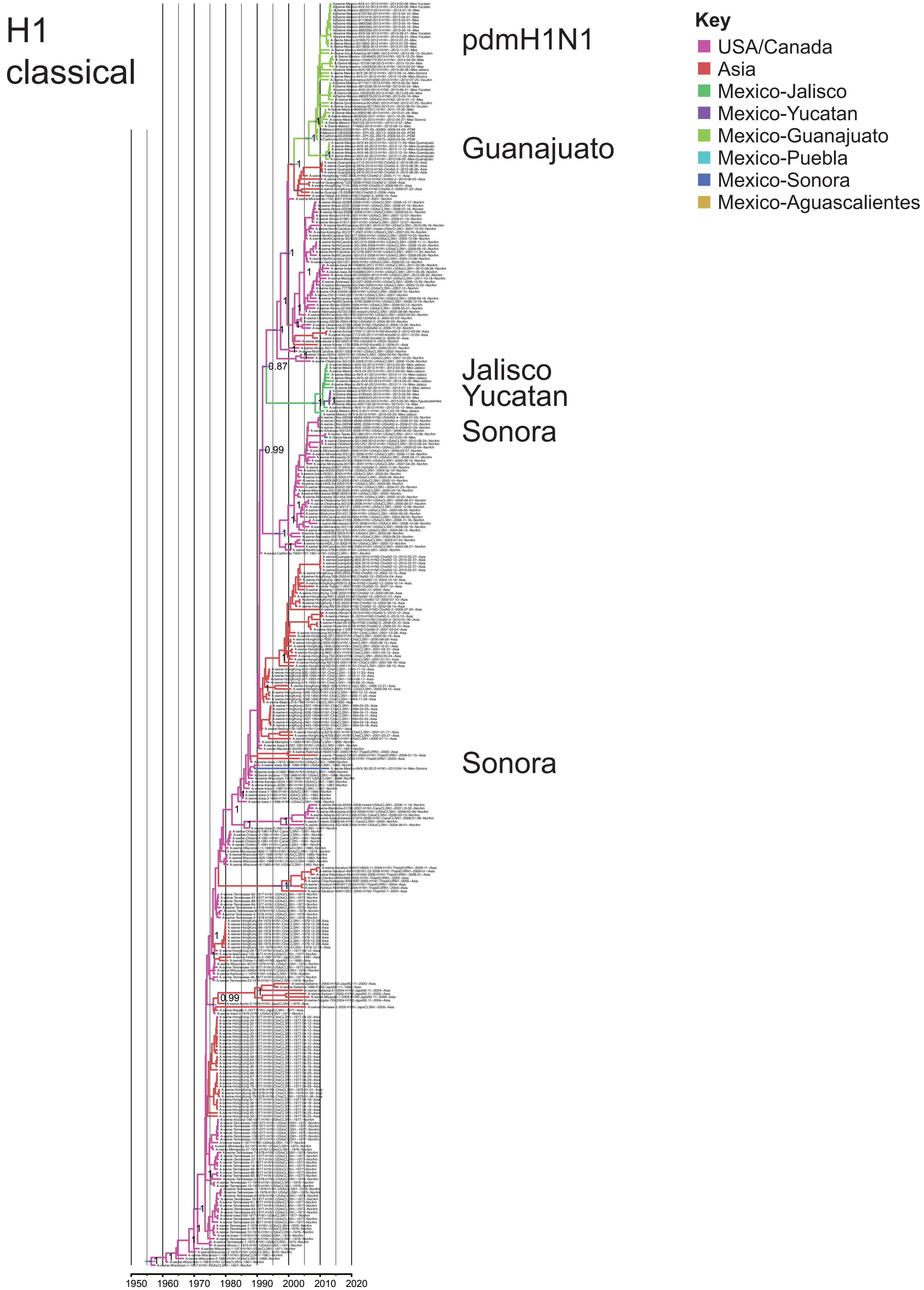

H1
classical
pdmH1N1
Guanajuato
Jalisco
Yucatan
Sonora
Sonora
Key
USA/Canada
Asia
Mexico-Jalisco
Mexico-Yucatan
Mexico-Guanajuato
Mexico-Puebla
Mexico-Sonora
Mexico-Aguascalientes
1950
1960
1970
1980
1990
2000
2010
2020
0.87
0.99
0.99

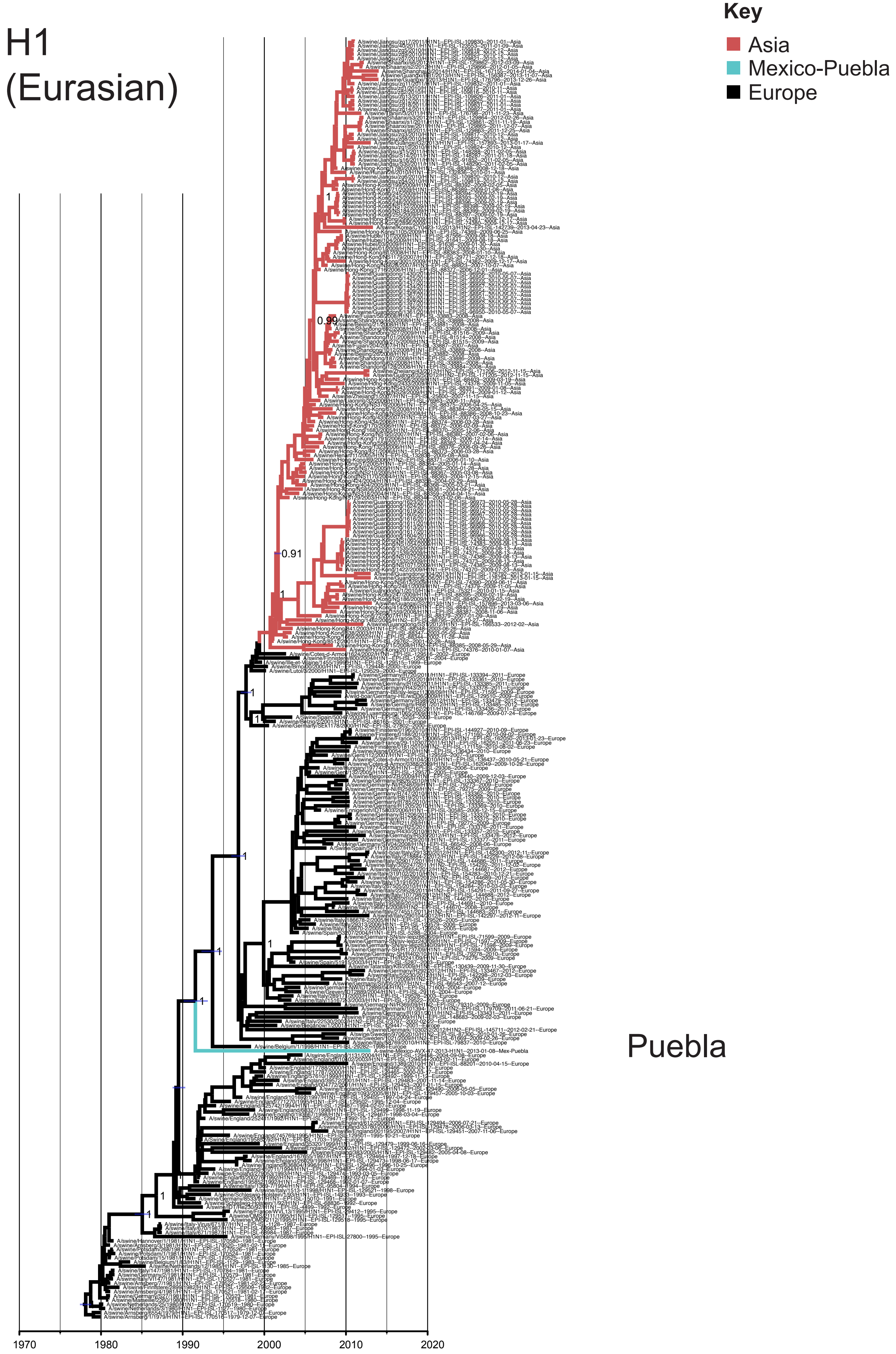

H1
(Eurasian)
Key
Asia
Mexico-Puebla
Europe
Puebla
1970
1980
1990
2000
2010
2020

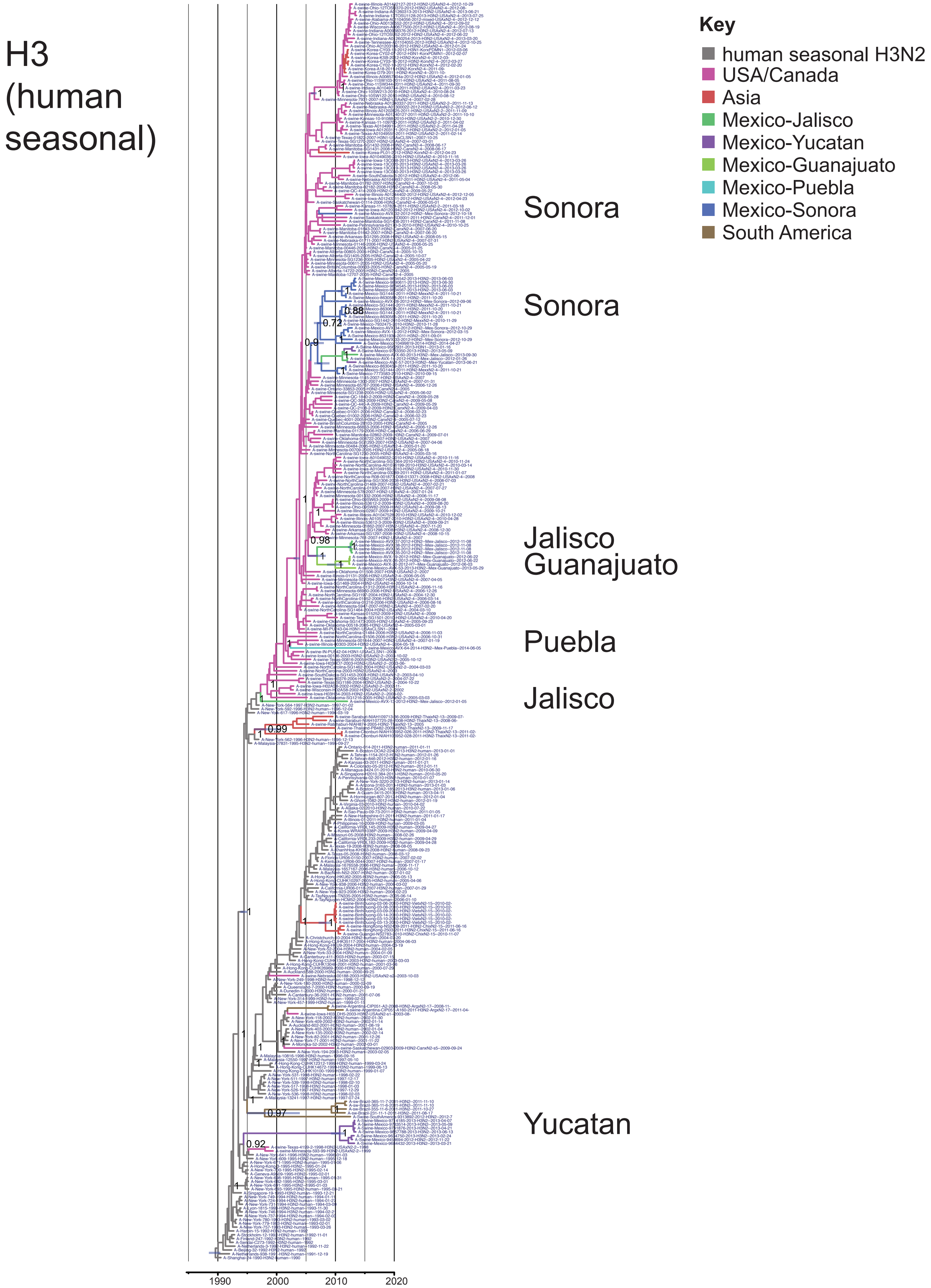

H3 (human seasonal)
Key
human seasonal H3N2
USA/Canada
Asia
Mexico-Jalisco
Mexico-Yucatan
Mexico-Guanajuato
Mexico-Puebla
Mexico-Sonora
South America
Sonora
Sonora
Jalisco
Guanajuato
Puebla
Jalisco
Yucatan
1990
2000
2010
2020

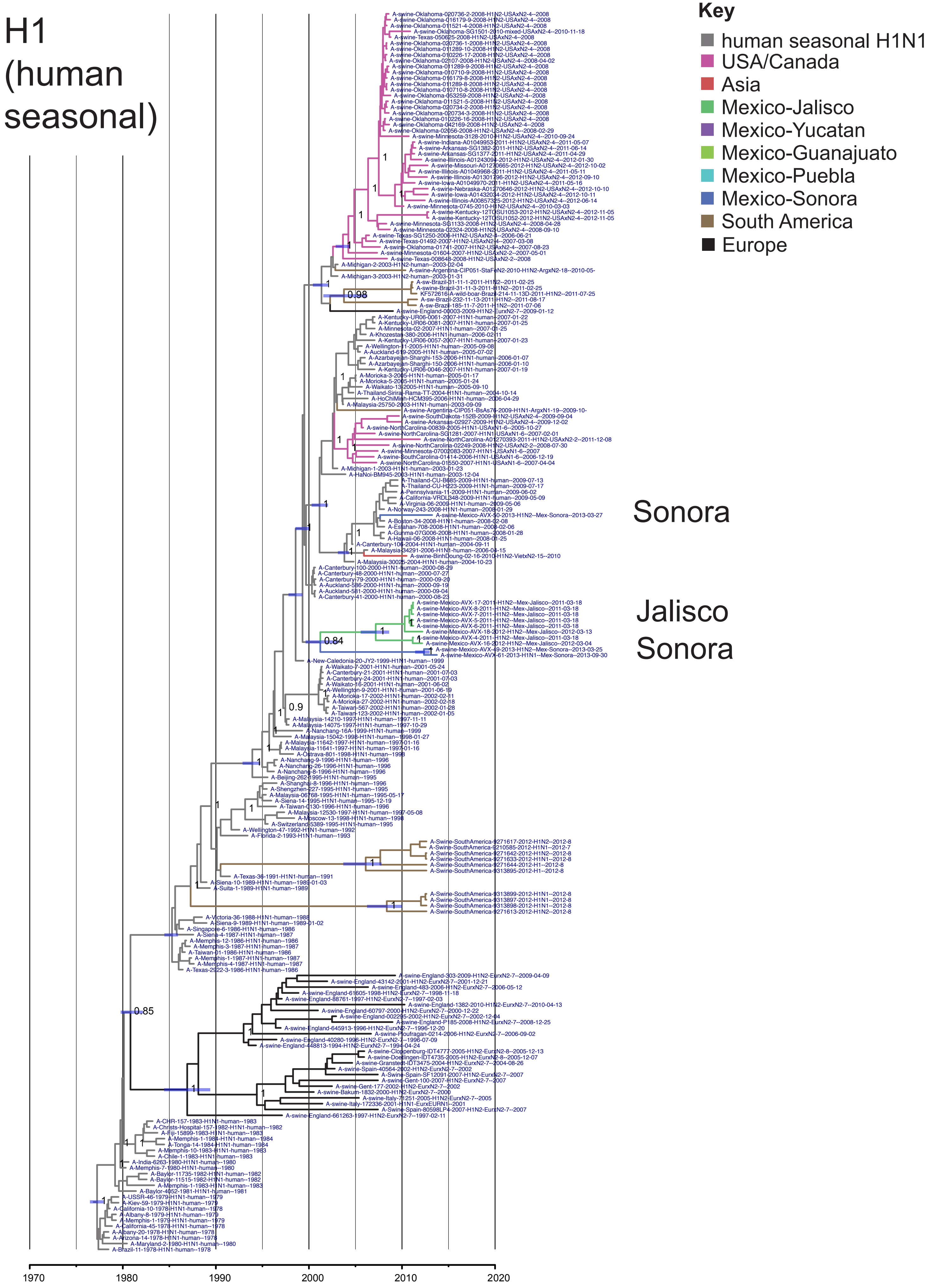

H1 (human seasonal)
Key
human seasonal H1N1
USA/Canada
Asia
Mexico-Jalisco
Mexico-Yucatan
Mexico-Guanajuato
Mexico-Puebla
Mexico-Sonora
South America
Europe
Sonora
Jalisco
Sonora
1970
1980
1990
2000
2010
2020

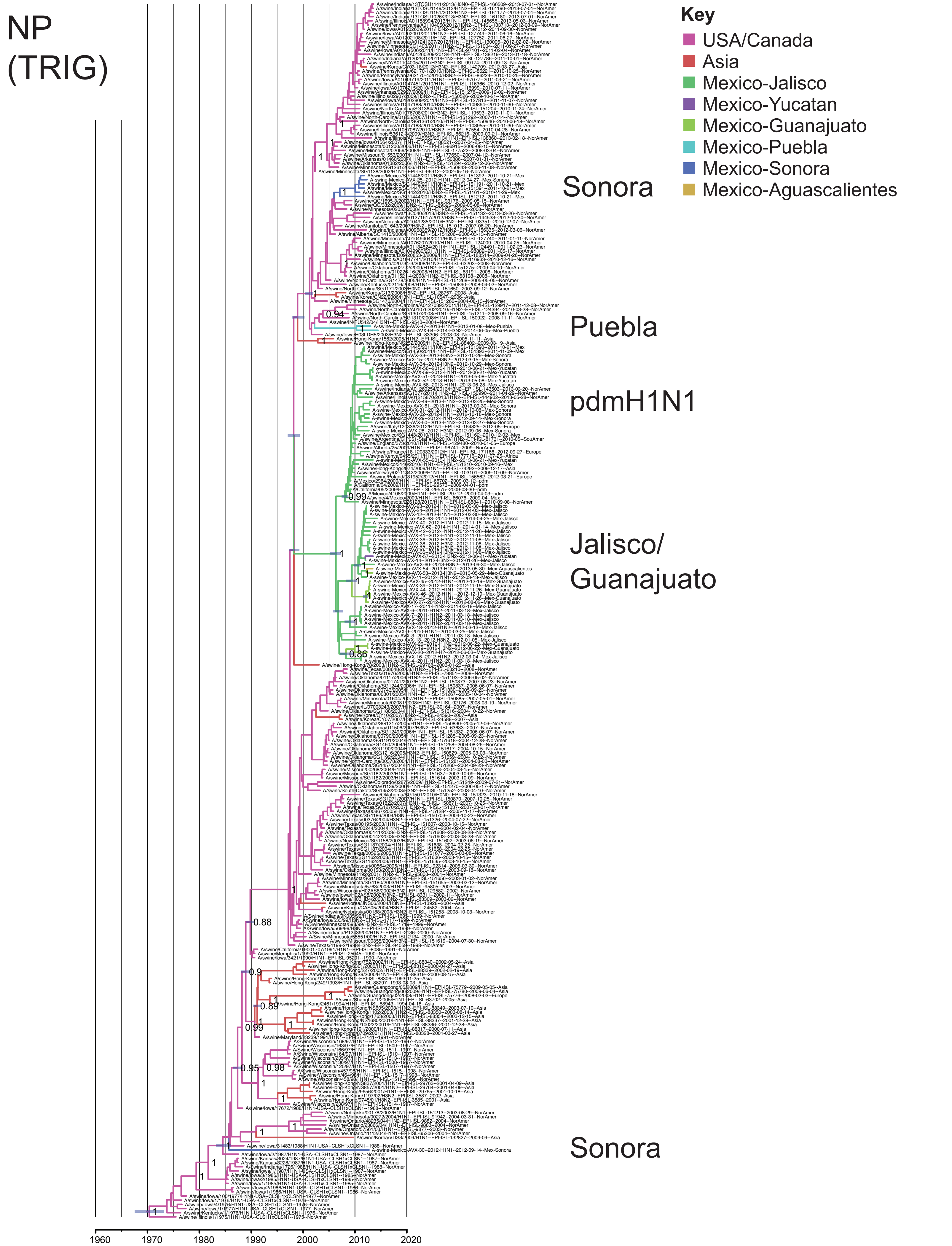

NP
(TRIG)
Key
USA/Canada
Asia
Mexico-Jalisco
Mexico-Yucatan
Mexico-Guanajuato
Mexico-Puebla
Mexico-Sonora
Mexico-Aguascalientes
Sonora
Puebla
pdmH1N1
Jalisco/
Guanajuato
Sonora
1960
1970
1980
1990
2000
2010
2020

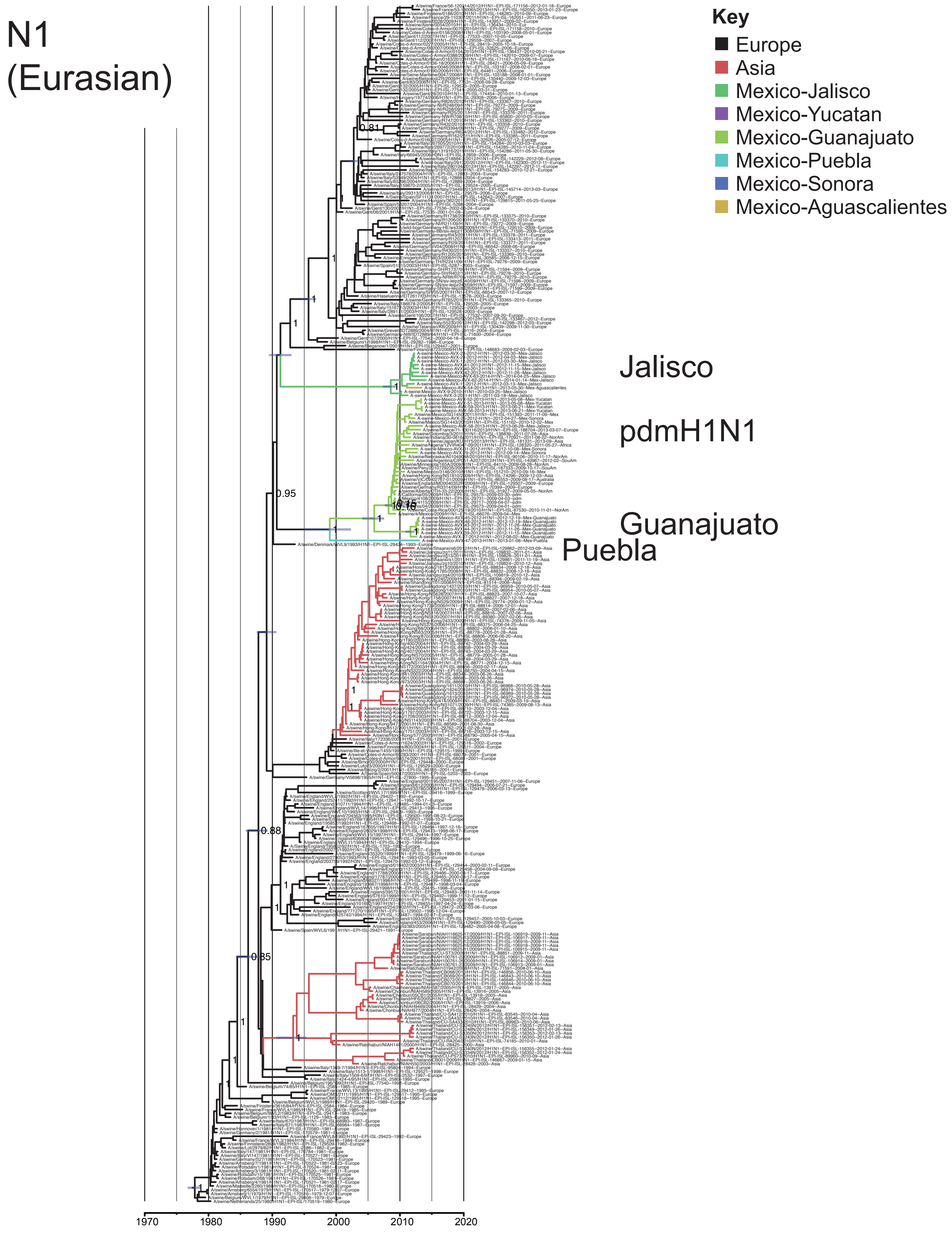
N1
(Eurasian)
Key
Europe
Asia
Mexico-Jalisco
Mexico-Yucatan
Mexico-Guanajuato
Mexico-Puebla
Mexico-Sonora
Mexico-Aguascalientes
Jalisco
pdmH1N1
Guanajuato
Puebla
1970
1980
1990
2000
2010
2020

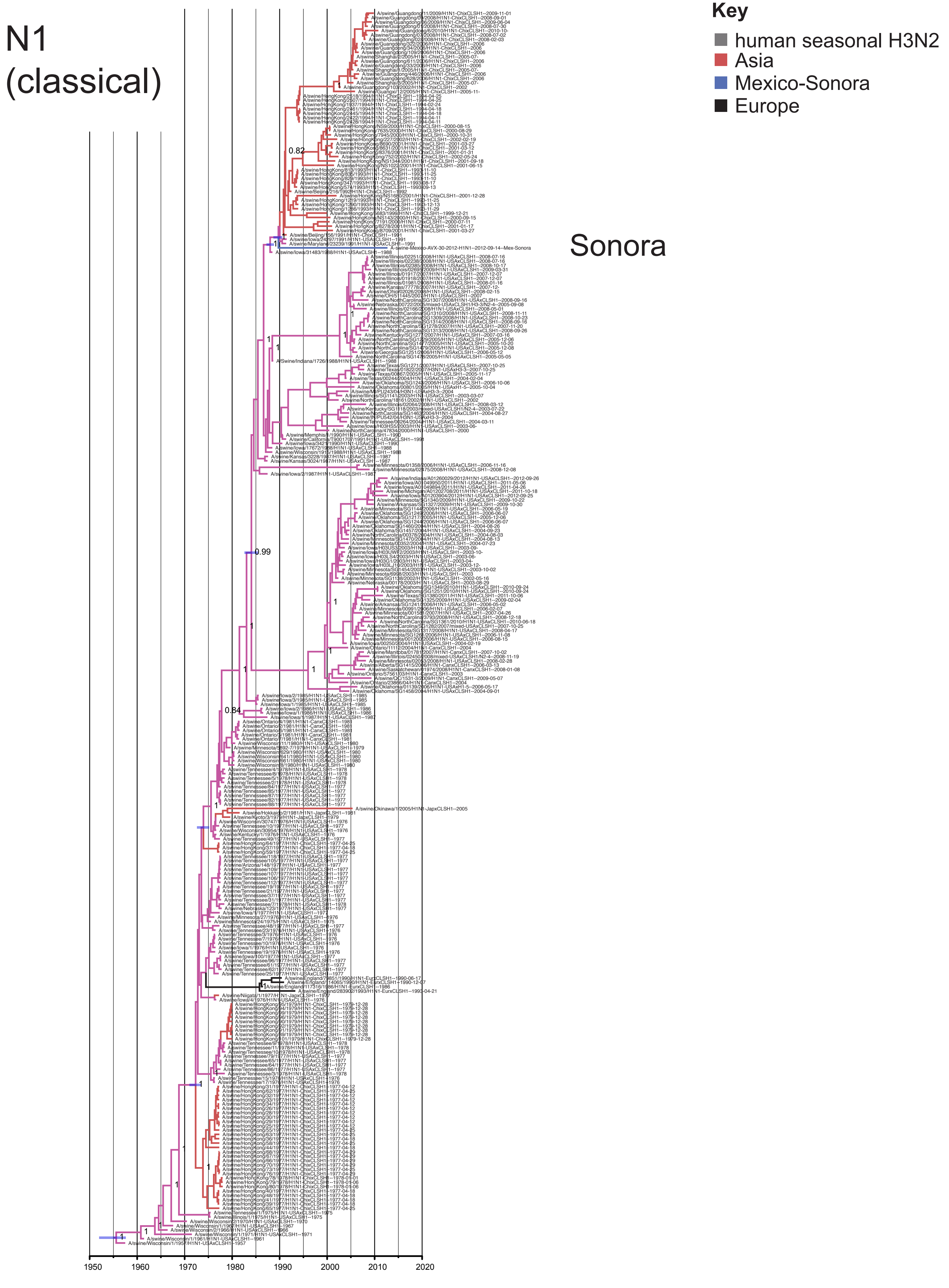

N1
(classical)
Key
human seasonal H3N2
Asia
Mexico-Sonora
Europe
Sonora
1950
1960
1970
1980
1990
2000
2010
2020

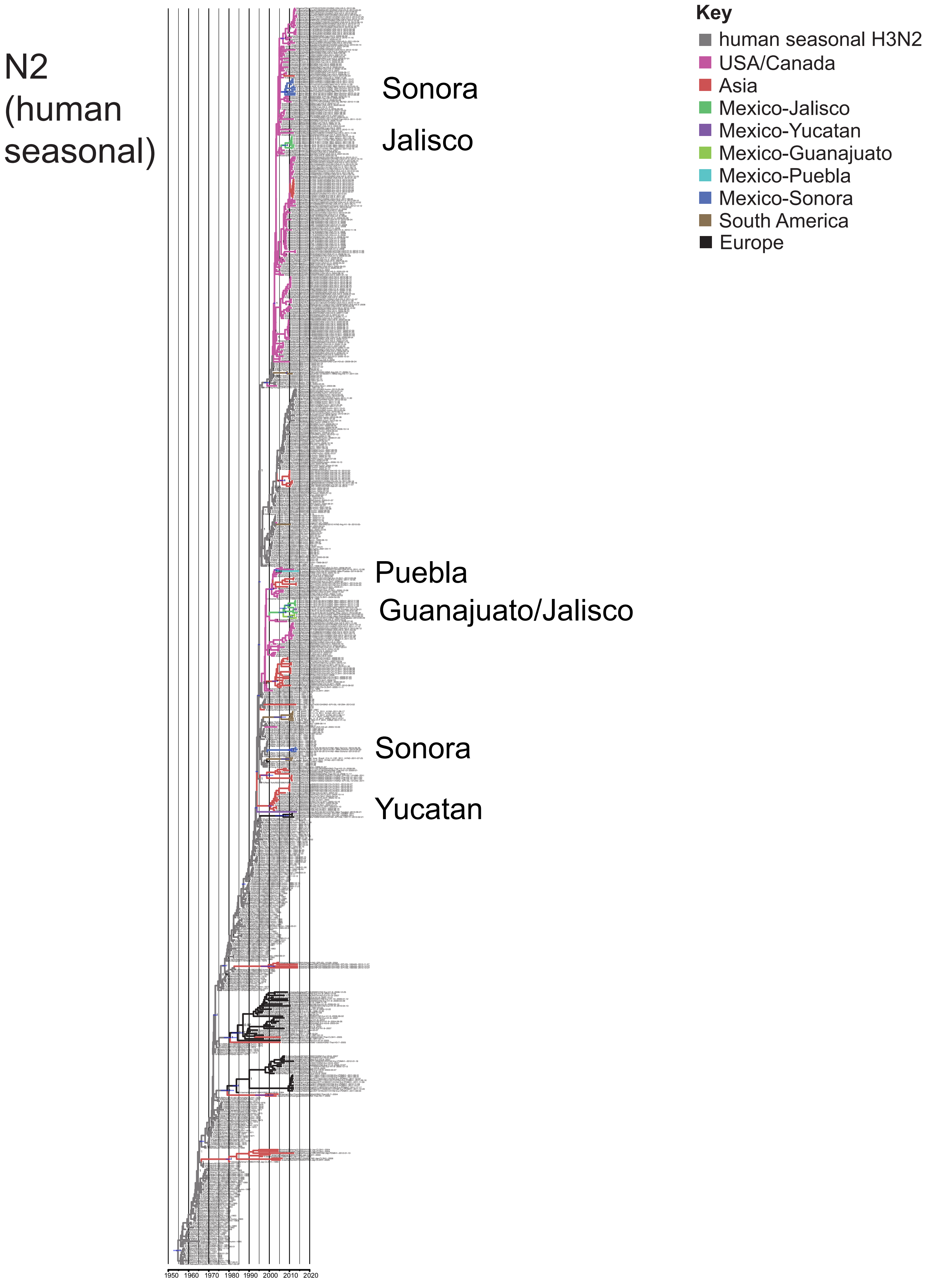

N2 (human seasonal)
Sonora
Jalisco
Puebla
Guanajuato/Jalisco
Sonora
Yucatan
1950
1960
1970
1980
1990
2000
2010
2020
Key
human seasonal H3N2
USA/Canada
Asia
Mexico-Jalisco
Mexico-Yucatan
Mexico-Guanajuato
Mexico-Puebla
Mexico-Sonora
South America
Europe

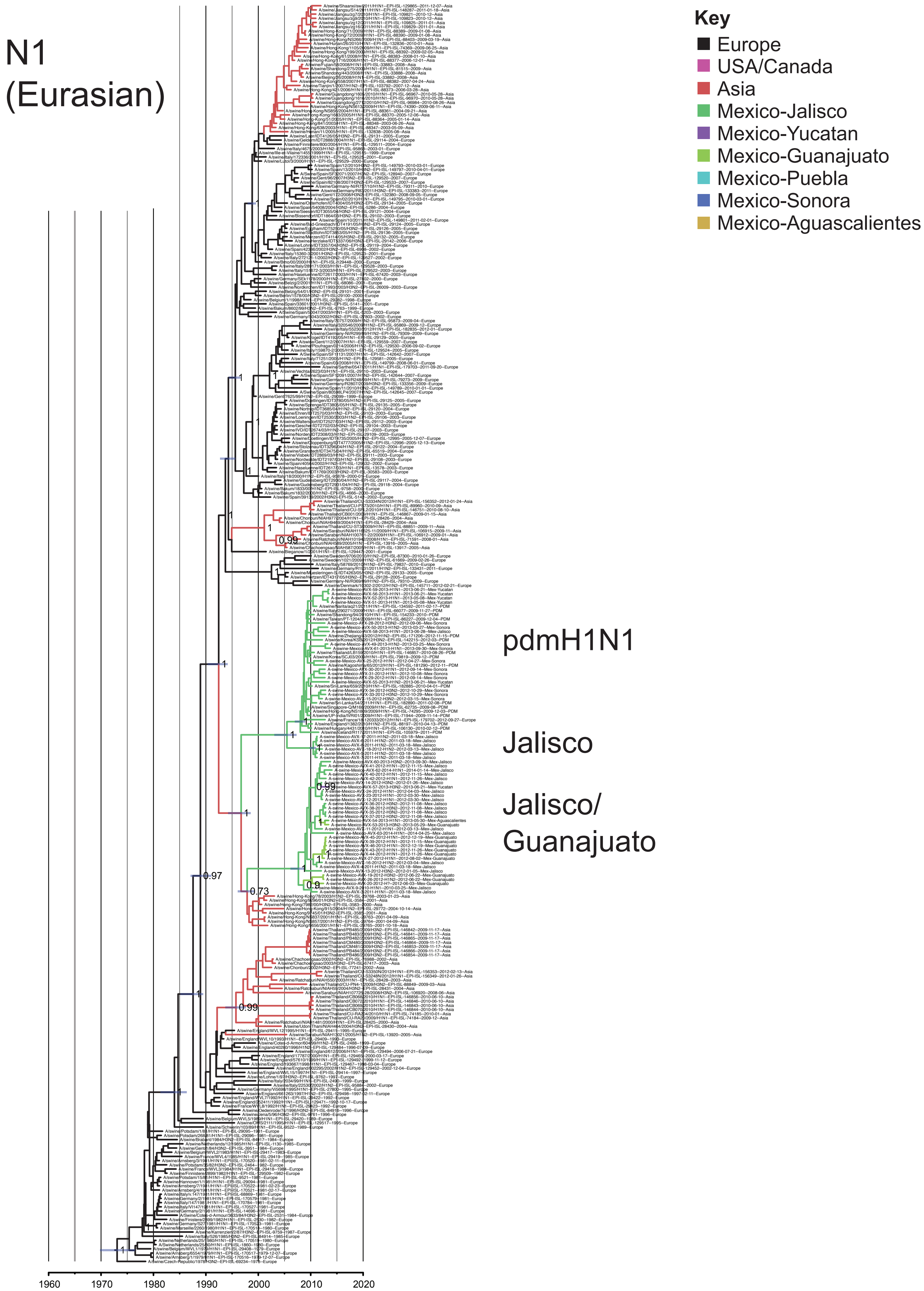
N1
(Eurasian)
Key
Europe
USA/Canada
Asia
Mexico-Jalisco
Mexico-Yucatan
Mexico-Guanajuato
Mexico-Puebla
Mexico-Sonora
Mexico-Aguascalientes
pdmH1N1
Jalisco
Jalisco/
Guanajuato
1960
1970
1980
1990
2000
2010
2020

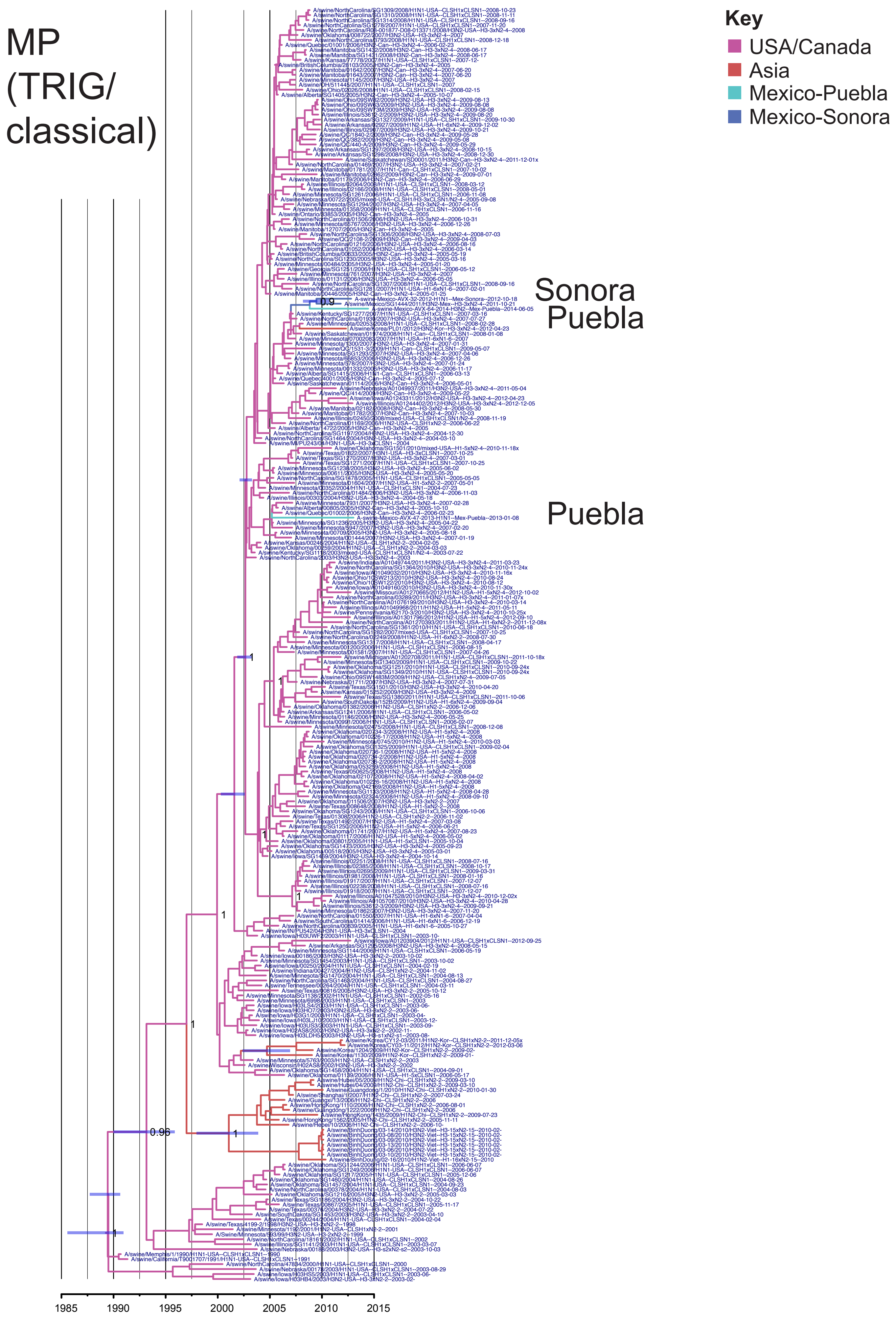
MP (TRIG/ classical)
Key
USA/Canada
Asia
Mexico-Puebla
Mexico-Sonora
Sonora
Puebla
Puebla
0.9
0.96
1
1985
1990
1995
2000
2005
2010
2015

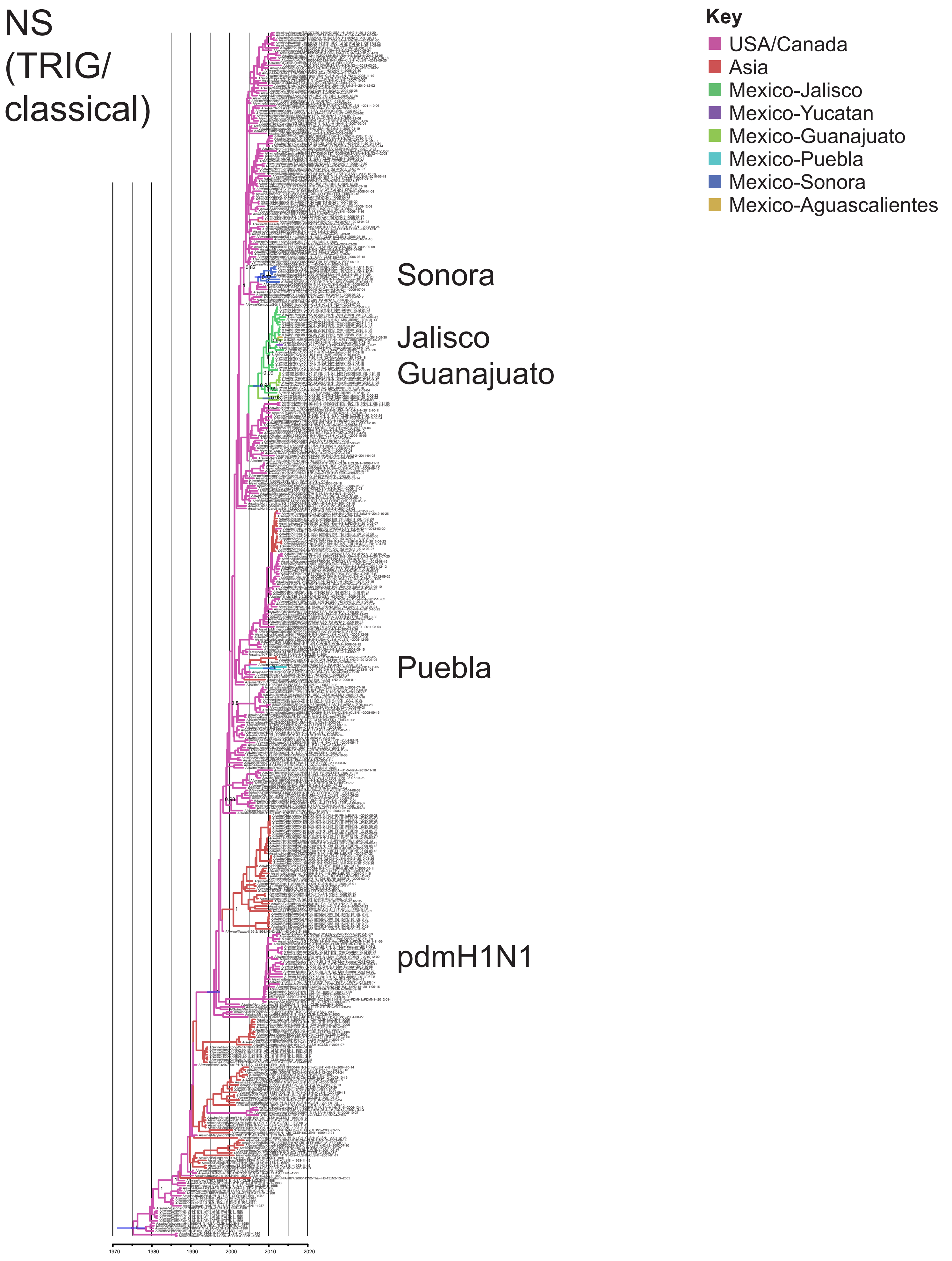
NS
(TRIG/
classical)
Key
USA/Canada
Asia
Mexico-Jalisco
Mexico-Yucatan
Mexico-Guanajuato
Mexico-Puebla
Mexico-Sonora
Mexico-Aguascalientes
Sonora
Jalisco
Guanajuato
Puebla
pdmH1N1
1970
1980
1990
2000
2010
2020
