# Supplementary material for: Origins of the 2009 H1N1 influenza pandemic in swine in Mexico
Source: eLife. 2016 Jun 28;5:e16777. doi: 10.7554/eLife.16777 (PMC4957980; doi:10.7554/eLife.16777)
Supplement: Figure 3—source data 2. — Evolutionary relationships of pdmH1N1 and swIAVs sampled in Mexico and globally for the eight segments of the IAV genome. Phylogenies and color scheme are similar to Figure 3, except the trees are inferred using maximum likelihood methods and all horizontal branch lengths are drawn to scale (nucleotide substitutions per site). Trees are midpoint rooted for clarity and bootstrap values >70 are provided for key nodes. DOI: http://dx.doi.org/10.7554/eLife.16777.009 [file elife-16777-fig3-data2.pdf]

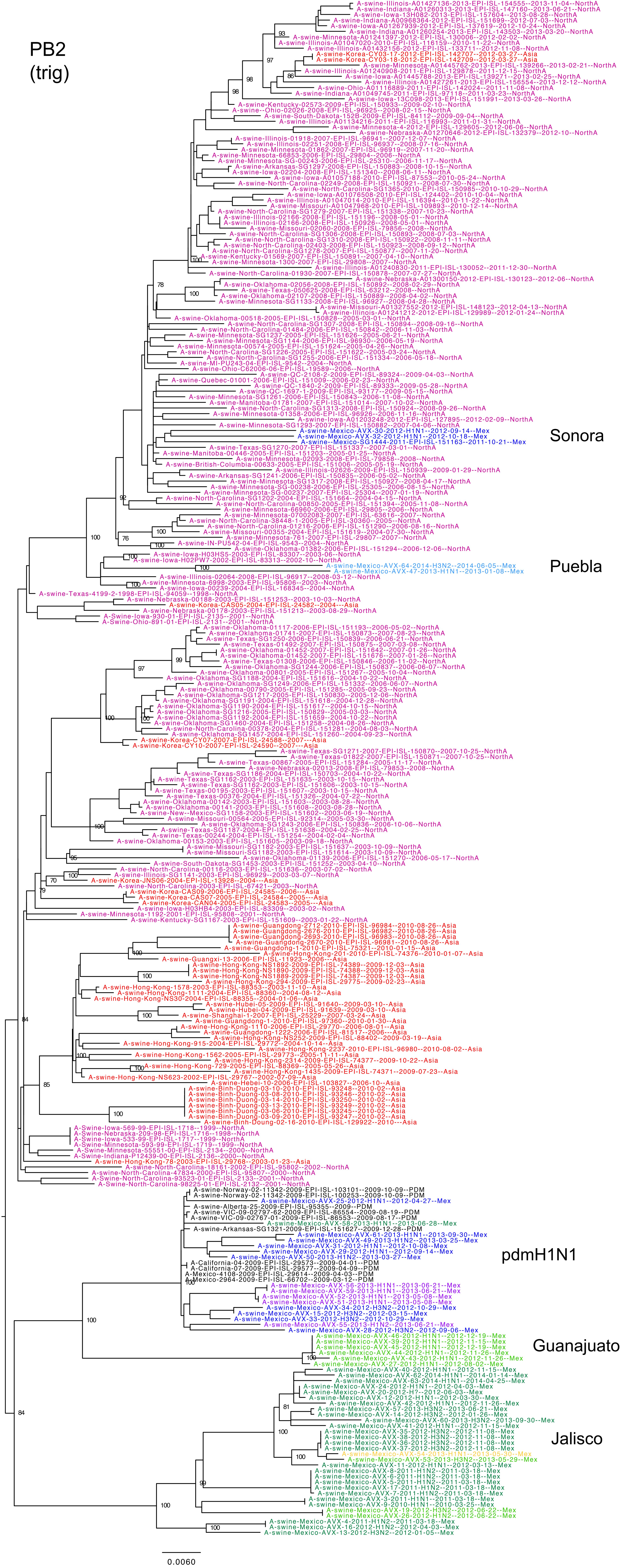
PB2
(trig)
Sonora
Puebla
pdmH1N1
Guanajuato
Jalisco
0.0060

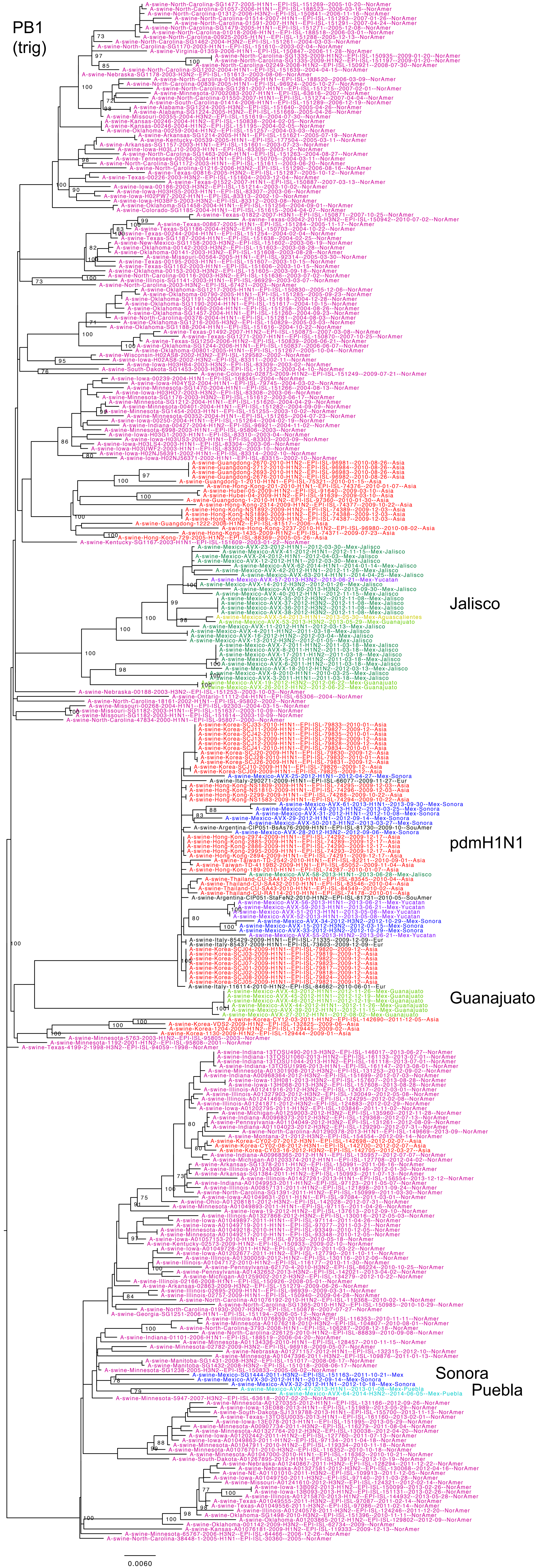
PB1
(trig)
Jalisco
pdmH1N1
Guanajuato
Sonora
Puebla
0.0060

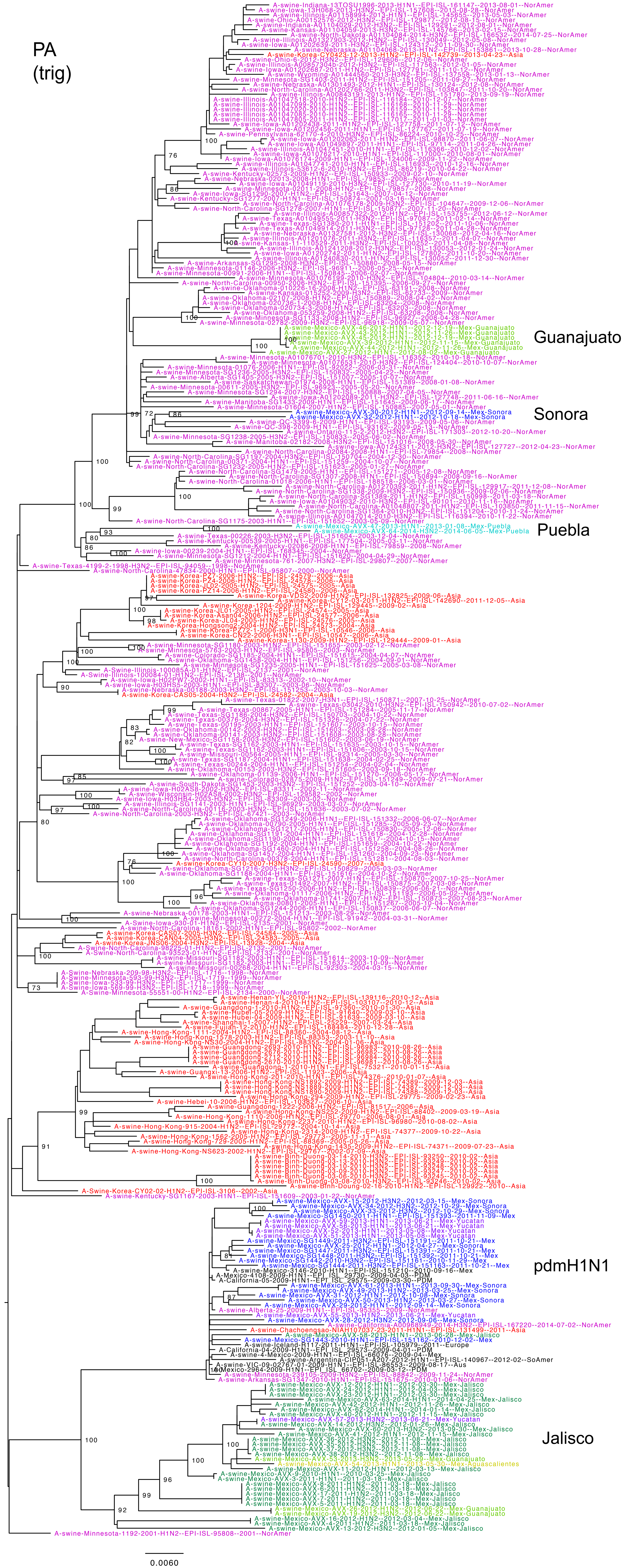

PA
(trig)
Guanajuato
Sonora
Puebla
pdmH1N1
Jalisco
0.0060

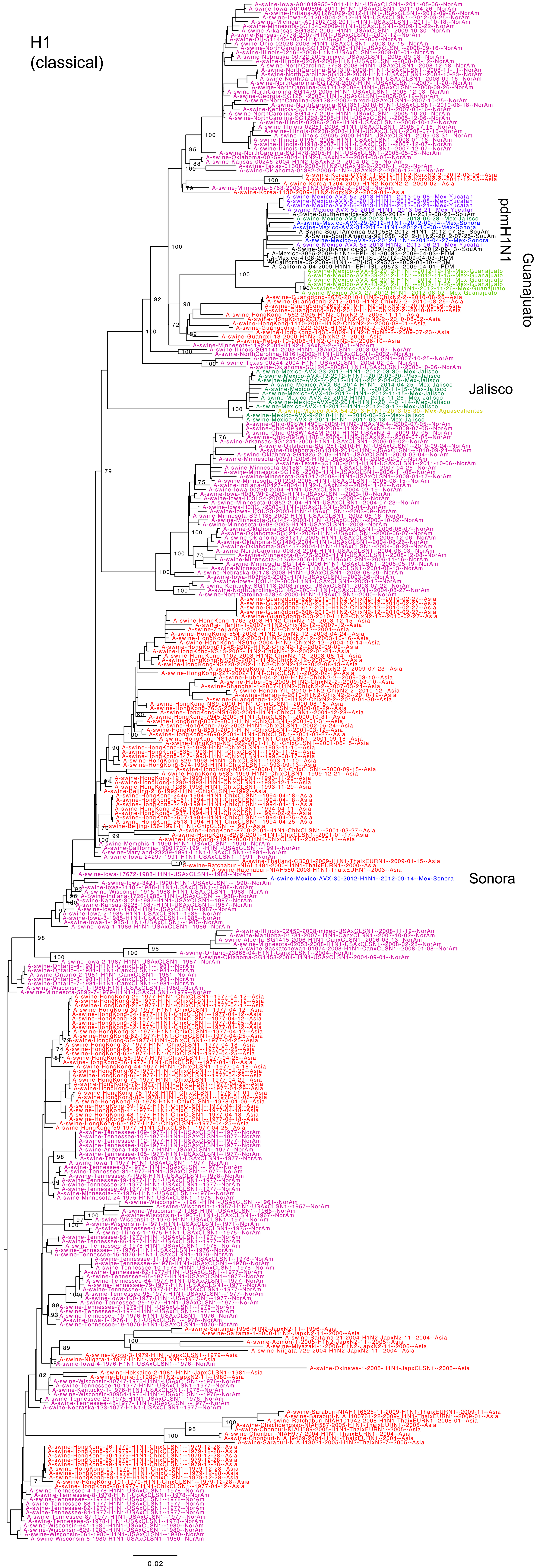
H1
(classical)
pdmH1N1
Guanajuato
Jalisco
Sonora
0.02

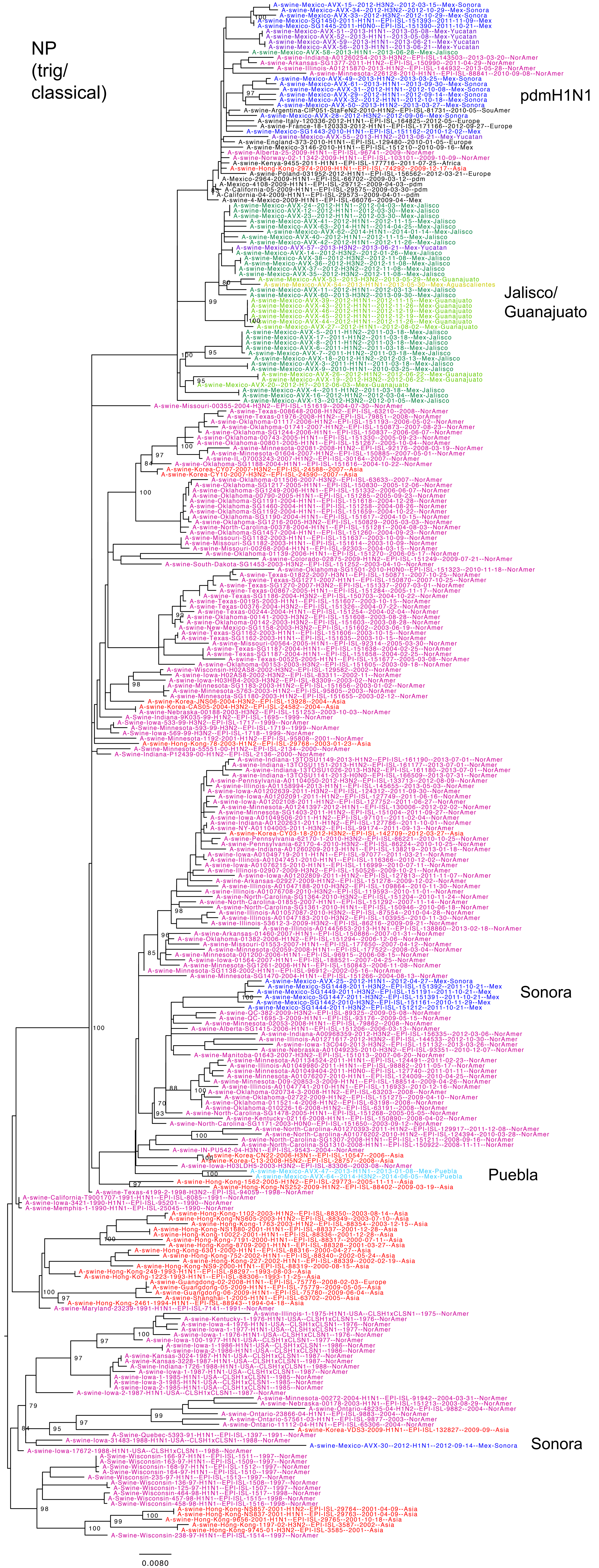
NP
(trig/
classical)
pdmH1N1
Jalisco/
Guanajuato
Sonora
Puebla
Sonora
0.0080

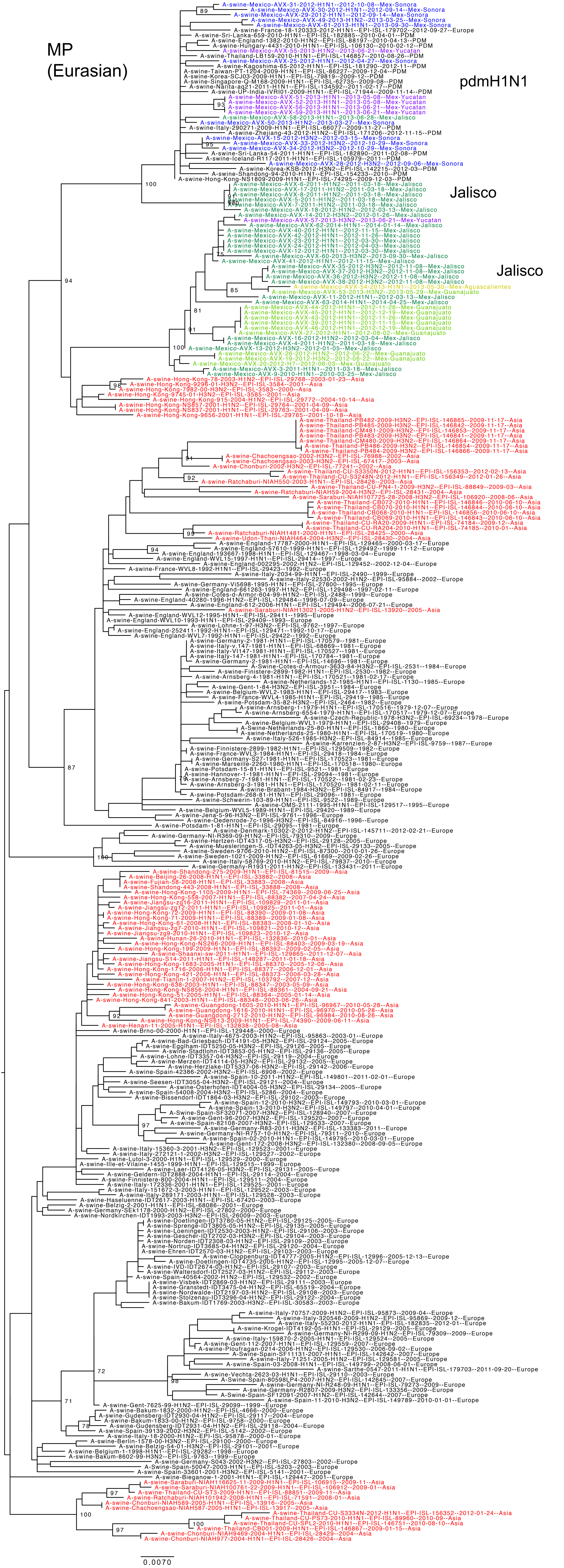

MP (Eurasian)
pdmH1N1
Jalisco
Jalisco
0.0070

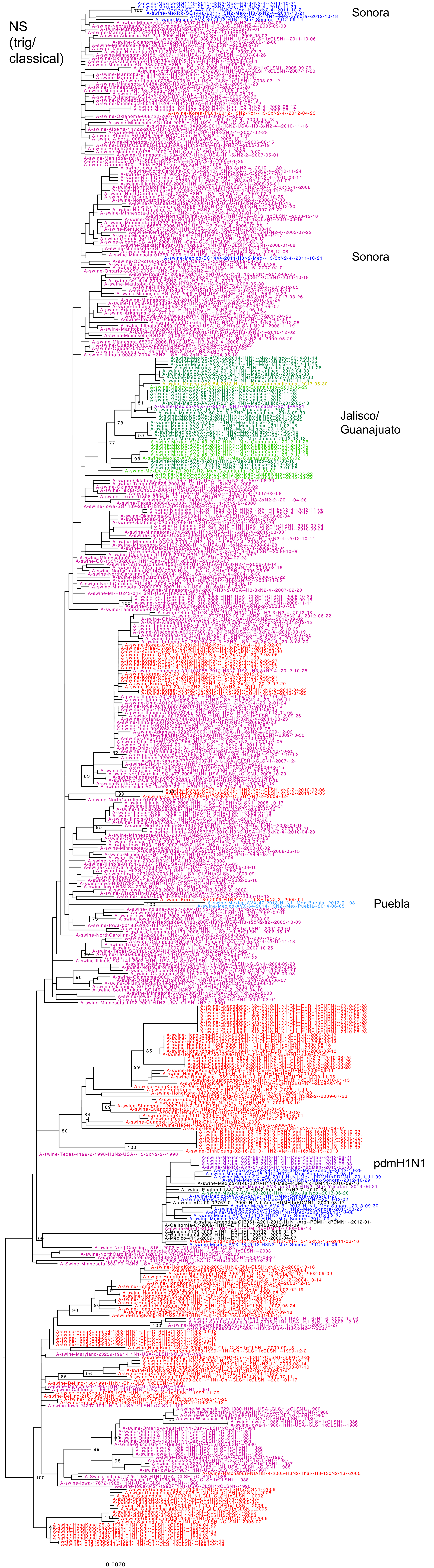
NS
(trig/
classical)
Sonora
Sonora
Jalisco/
Guanajuato
Puebla
pdmH1N1
0.0070
