# Supplementary material for: Origins of the 2009 H1N1 influenza pandemic in swine in Mexico
Source: eLife. 2016 Jun 28;5:e16777. doi: 10.7554/eLife.16777 (PMC4957980; doi:10.7554/eLife.16777)
Supplement: Figure 3—source data 3. — Similar phylogenies as presented in Figure 3—source data 1, but in this case Mexican swine viruses are specified by genotype instead of geography. Non-Mexican viruses remain specified by geographical location. Markov rewards representing the time spent in a genotype between state transitions are provided in the upper right. Genotype 1, which is similar to pdmH1N1 (Figure 2), is shaded gold. DOI: http://dx.doi.org/10.7554/eLife.16777.010 [file elife-16777-fig3-data3.pdf]

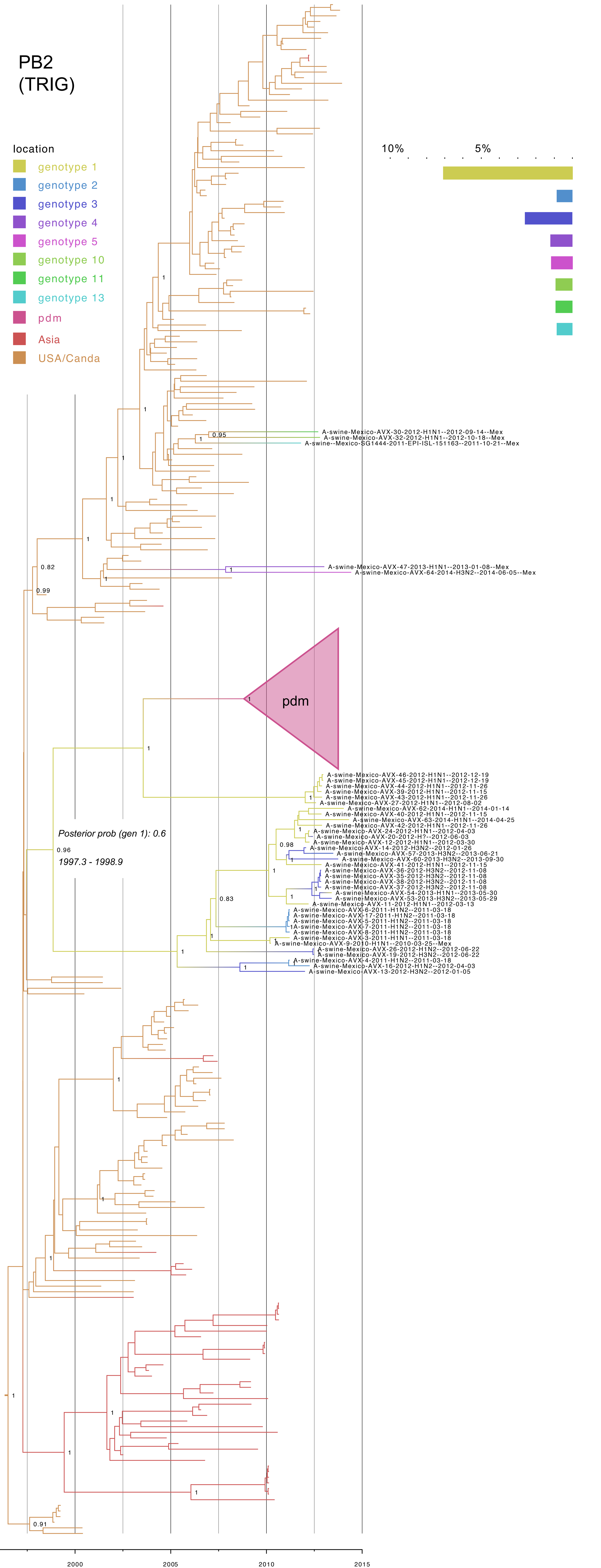
PB2
(TRIG)
location
genotype 1
genotype 2
genotype 3
genotype 4
genotype 5
genotype 10
genotype 11
genotype 13
pdm
Asia
USA/Canda
10%
5%
A-swine-Mexico-AVX-30-2012-H1N1--2012-09-14--Mex
A-swine-Mexico-AVX-32-2012-H1N1--2012-10-18--Mex
A-swine--Mexico-SG1444-2011-EPI-ISL-151163--2011-10-21--Mex
A-swine-Mexico-AVX-47-2013-H1N1--2013-01-08--Mex
A-swine-Mexico-AVX-64-2014-H3N2--2014-06-05--Mex
pdm
A-swine-Mexico-AVX-46-2012-H1N1--2012-12-19
A-swine-Mexico-AVX-45-2012-H1N1--2012-12-19
A-swine-Mexico-AVX-44-2012-H1N1--2012-11-26
A-swine-Mexico-AVX-39-2012-H1N1--2012-11-15
A-swine-Mexico-AVX-43-2012-H1N1--2012-11-26
A-swine-Mexico-AVX-27-2012-H1N1--2012-08-02
A-swine-Mexico-AVX-62-2014-H1N1--2014-01-14
A-swine-Mexico-AVX-40-2012-H1N1--2012-11-15
A-swine-Mexico-AVX-63-2014-H1N1--2014-04-25
A-swine-Mexico-AVX-42-2012-H1N1--2012-11-26
A-swine-Mexico-AVX-24-2012-H1N1--2012-04-03
A-swine-Mexico-AVX-20-2012-H?--2012-06-03
A-swine-Mexico-AVX-12-2012-H1N1--2012-03-30
A-swine-Mexico-AVX-14-2012-H3N2--2012-01-26
A-swine-Mexico-AVX-57-2013-H3N2--2013-06-21
A-swine-Mexico-AVX-60-2013-H3N2--2013-09-30
A-swine-Mexico-AVX-41-2012-H1N1--2012-11-15
A-swine-Mexico-AVX-36-2012-H3N2--2012-11-08
A-swine-Mexico-AVX-35-2012-H3N2--2012-11-08
A-swine-Mexico-AVX-38-2012-H3N2--2012-11-08
A-swine-Mexico-AVX-37-2012-H3N2--2012-11-08
A-swine-Mexico-AVX-54-2013-H1N1--2013-05-30
A-swine-Mexico-AVX-53-2013-H3N2--2013-05-29
A-swine-Mexico-AVX-11-2012-H1N1--2012-03-13
A-swine-Mexico-AVX-6-2011-H1N2--2011-03-18
A-swine-Mexico-AVX-17-2011-H1N2--2011-03-18
A-swine-Mexico-AVX-5-2011-H1N2--2011-03-18
A-swine-Mexico-AVX-7-2011-H1N2--2011-03-18
A-swine-Mexico-AVX-8-2011-H1N2--2011-03-18
A-swine-Mexico-AVX-3-2011-H1N1--2011-03-18
A-swine-Mexico-AVX-9-2010-H1N1--2010-03-25--Mex
A-swine-Mexico-AVX-26-2012-H1N2--2012-06-22
A-swine-Mexico-AVX-19-2012-H3N2--2012-06-22
A-swine-Mexico-AVX-4-2011-H1N2--2011-03-18
A-swine-Mexico-AVX-16-2012-H1N2--2012-04-03
A-swine-Mexico-AVX-13-2012-H3N2--2012-01-05
Posterior prob (gen 1): 0.6
0.96
1997.3 - 1998.9
2000
2005
2010
2015

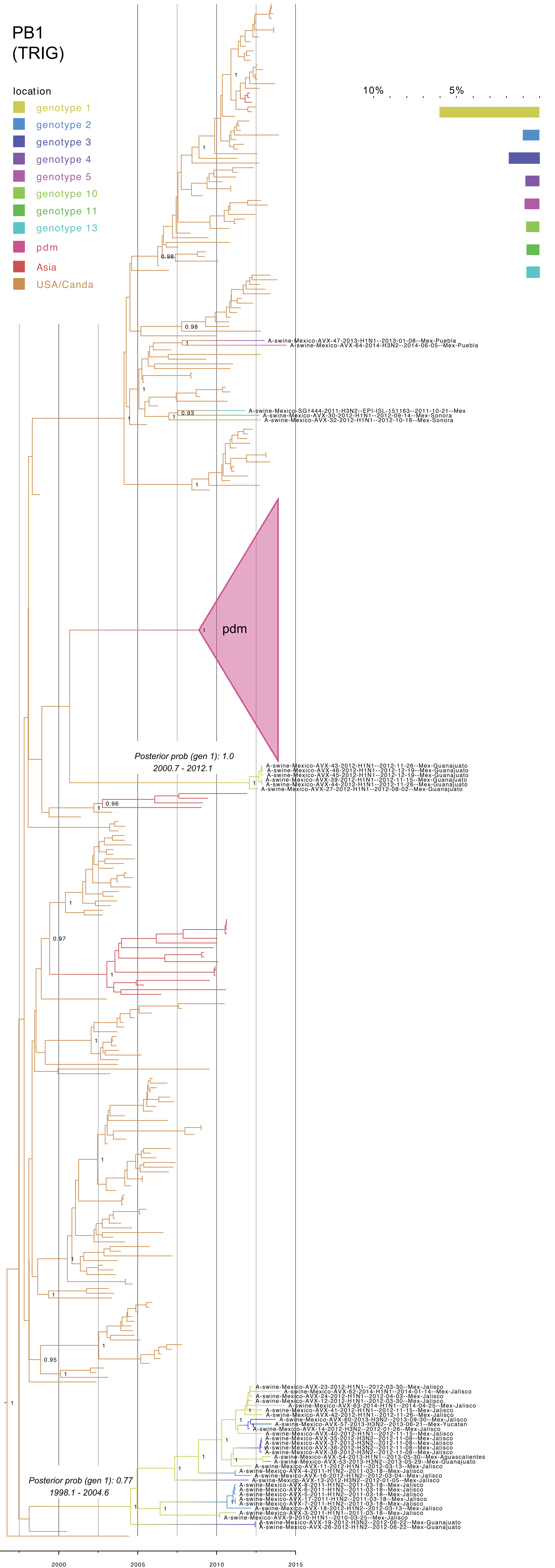
PB1
(TRIG)
location
genotype 1
genotype 2
genotype 3
genotype 4
genotype 5
genotype 10
genotype 11
genotype 13
pdm
Asia
USA/Canda
10%
5%
A-swine-Mexico-AVX-47-2013-H1N1--2013-01-08--Mex-Puebla
A-swine-Mexico-AVX-64-2014-H3N2--2014-06-05--Mex-Puebla
A-swine-Mexico-SG1444-2011-H3N2--EPI-ISL-151163--2011-10-21--Mex
A-swine-Mexico-AVX-30-2012-H1N1--2012-09-14--Mex-Sonora
A-swine-Mexico-AVX-32-2012-H1N1--2012-10-18--Mex-Sonora
pdm
Posterior prob (gen 1): 1.0
2000.7 - 2012.1
A-swine-Mexico-AVX-43-2012-H1N1--2012-11-26--Mex-Guanajuato
A-swine-Mexico-AVX-46-2012-H1N1--2012-12-19--Mex-Guanajuato
A-swine-Mexico-AVX-45-2012-H1N1--2012-12-19--Mex-Guanajuato
A-swine-Mexico-AVX-39-2012-H1N1--2012-11-15--Mex-Guanajuato
A-swine-Mexico-AVX-44-2012-H1N1--2012-11-26--Mex-Guanajuato
A-swine-Mexico-AVX-27-2012-H1N1--2012-08-02--Mex-Guanajuato
A-swine-Mexico-AVX-23-2012-H1N1--2012-03-30--Mex-Jalisco
A-swine-Mexico-AVX-62-2014-H1N1--2014-01-14--Mex-Jalisco
A-swine-Mexico-AVX-24-2012-H1N1--2012-04-03--Mex-Jalisco
A-swine-Mexico-AVX-12-2012-H1N1--2012-03-30--Mex-Jalisco
A-swine-Mexico-AVX-63-2014-H1N1--2014-04-25--Mex-Jalisco
A-swine-Mexico-AVX-41-2012-H1N1--2012-11-15--Mex-Jalisco
A-swine-Mexico-AVX-42-2012-H1N1--2012-11-26--Mex-Jalisco
A-swine-Mexico-AVX-60-2013-H3N2--2013-09-30--Mex-Jalisco
A-swine-Mexico-AVX-57-2013-H3N2--2013-06-21--Mex-Yucatan
A-swine-Mexico-AVX-14-2012-H3N2--2012-01-26--Mex-Jalisco
A-swine-Mexico-AVX-40-2012-H1N1--2012-11-15--Mex-Jalisco
A-swine-Mexico-AVX-35-2012-H3N2--2012-11-08--Mex-Jalisco
A-swine-Mexico-AVX-37-2012-H3N2--2012-11-08--Mex-Jalisco
A-swine-Mexico-AVX-36-2012-H3N2--2012-11-08--Mex-Jalisco
A-swine-Mexico-AVX-38-2012-H3N2--2012-11-08--Mex-Jalisco
A-swine-Mexico-AVX-54-2013-H1N1--2013-05-30--Mex-Aguascalientes
A-swine-Mexico-AVX-53-2013-H3N2--2013-05-29--Mex-Guanajuato
A-swine-Mexico-AVX-11-2012-H1N1--2012-03-13--Mex-Jalisco
A-swine-Mexico-AVX-4-2011-H1N2--2011-03-18--Mex-Jalisco
A-swine-Mexico-AVX-16-2012-H1N2--2012-03-04--Mex-Jalisco
A-swine-Mexico-AVX-13-2012-H3N2--2012-01-05--Mex-Jalisco
A-swine-Mexico-AVX-8-2011-H1N2--2011-03-18--Mex-Jalisco
A-swine-Mexico-AVX-6-2011-H1N2--2011-03-18--Mex-Jalisco
A-swine-Mexico-AVX-5-2011-H1N2--2011-03-18--Mex-Jalisco
A-swine-Mexico-AVX-17-2011-H1N2--2011-03-18--Mex-Jalisco
A-swine-Mexico-AVX-7-2011-H1N2--2011-03-18--Mex-Jalisco
A-swine-Mexico-AVX-18-2012-H1N2--2012-03-13--Mex-Jalisco
A-swine-Mexico-AVX-3-2011-H1N1--2011-03-18--Mex-Jalisco
A-swine-Mexico-AVX-9-2010-H1N1--2010-03-25--Mex-Jalisco
A-swine-Mexico-AVX-19-2012-H3N2--2012-06-22--Mex-Guanajuato
A-swine-Mexico-AVX-26-2012-H1N2--2012-06-22--Mex-Guanajuato
Posterior prob (gen 1): 0.77
1998.1 - 2004.6
2000
2005
2010
2015

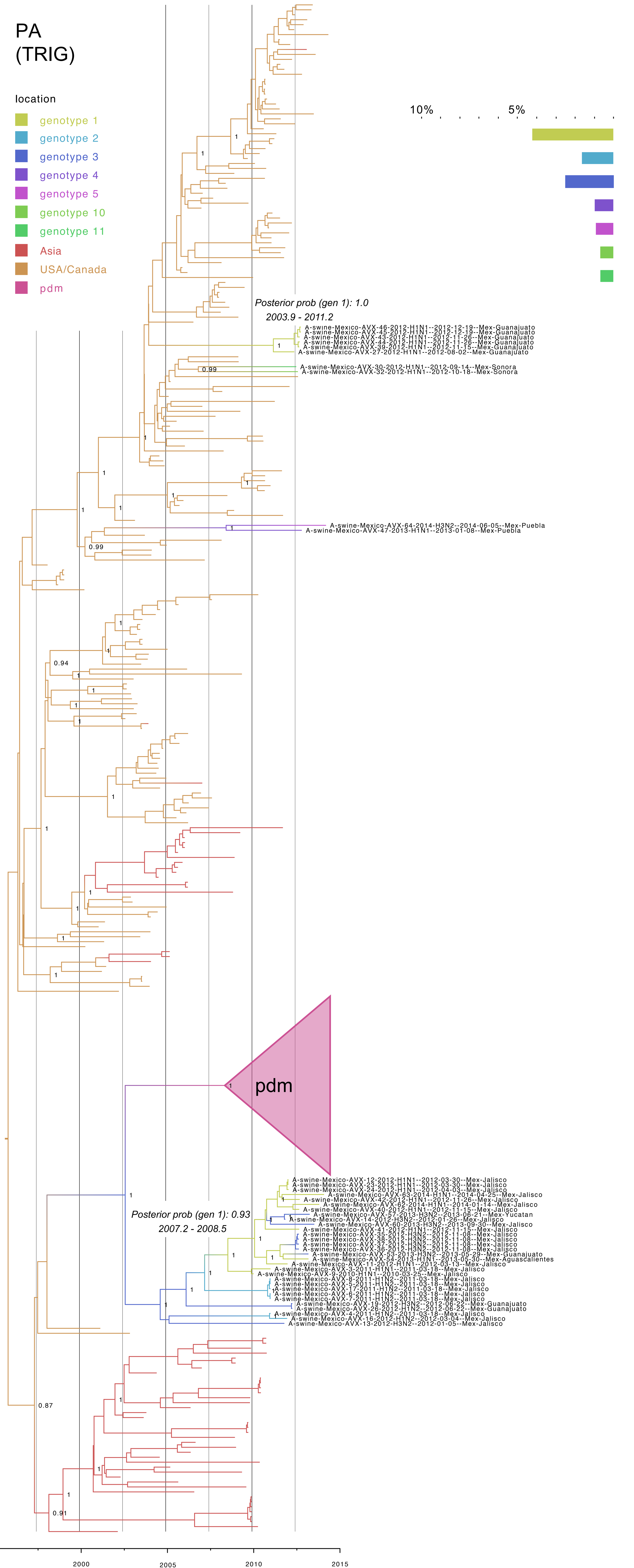
PA
(TRIG)
location
genotype 1
genotype 2
genotype 3
genotype 4
genotype 5
genotype 10
genotype 11
Asia
USA/Canada
pdm
10%
5%
Posterior prob (gen 1): 1.0
2003.9 - 2011.2
A-swine-Mexico-AVX-46-2012-H1N1--2012-12-19--Mex-Guanajuato
A-swine-Mexico-AVX-45-2012-H1N1--2012-12-19--Mex-Guanajuato
A-swine-Mexico-AVX-43-2012-H1N1--2012-11-26--Mex-Guanajuato
A-swine-Mexico-AVX-44-2012-H1N1--2012-11-26--Mex-Guanajuato
A-swine-Mexico-AVX-39-2012-H1N1--2012-11-15--Mex-Guanajuato
A-swine-Mexico-AVX-27-2012-H1N1--2012-08-02--Mex-Guanajuato
A-swine-Mexico-AVX-30-2012-H1N1--2012-09-14--Mex-Sonora
A-swine-Mexico-AVX-32-2012-H1N1--2012-10-18--Mex-Sonora
A-swine-Mexico-AVX-64-2014-H3N2--2014-06-05--Mex-Puebla
A-swine-Mexico-AVX-47-2013-H1N1--2013-01-08--Mex-Puebla
pdm
Posterior prob (gen 1): 0.93
2007.2 - 2008.5
A-swine-Mexico-AVX-12-2012-H1N1--2012-03-30--Mex-Jalisco
A-swine-Mexico-AVX-23-2012-H1N1--2012-03-30--Mex-Jalisco
A-swine-Mexico-AVX-24-2012-H1N1--2012-04-03--Mex-Jalisco
A-swine-Mexico-AVX-63-2014-H1N1--2014-04-25--Mex-Jalisco
A-swine-Mexico-AVX-42-2012-H1N1--2012-11-26--Mex-Jalisco
A-swine-Mexico-AVX-62-2014-H1N1--2014-01-14--Mex-Jalisco
A-swine-Mexico-AVX-40-2012-H1N1--2012-11-15--Mex-Jalisco
A-swine-Mexico-AVX-57-2013-H3N2--2013-06-21--Mex-Yucatan
A-swine-Mexico-AVX-14-2012-H3N2--2012-01-26--Mex-Jalisco
A-swine-Mexico-AVX-60-2013-H3N2--2013-09-30--Mex-Jalisco
A-swine-Mexico-AVX-41-2012-H1N1--2012-11-15--Mex-Jalisco
A-swine-Mexico-AVX-35-2012-H3N2--2012-11-08--Mex-Jalisco
A-swine-Mexico-AVX-38-2012-H3N2--2012-11-08--Mex-Jalisco
A-swine-Mexico-AVX-37-2012-H3N2--2012-11-08--Mex-Jalisco
A-swine-Mexico-AVX-36-2012-H3N2--2012-11-08--Mex-Jalisco
A-swine-Mexico-AVX-53-2013-H3N2--2013-05-29--Mex-Guanajuato
A-swine-Mexico-AVX-54-2013-H1N1--2013-05-30--Mex-Aguascalientes
A-swine-Mexico-AVX-11-2012-H1N1--2012-03-13--Mex-Jalisco
A-swine-Mexico-AVX-3-2011-H1N1--2011-03-18--Mex-Jalisco
A-swine-Mexico-AVX-9-2010-H1N1--2010-03-25--Mex-Jalisco
A-swine-Mexico-AVX-8-2011-H1N2--2011-03-18--Mex-Jalisco
A-swine-Mexico-AVX-5-2011-H1N2--2011-03-18--Mex-Jalisco
A-swine-Mexico-AVX-17-2011-H1N2--2011-03-18--Mex-Jalisco
A-swine-Mexico-AVX-6-2011-H1N2--2011-03-18--Mex-Jalisco
A-swine-Mexico-AVX-7-2011-H1N2--2011-03-18--Mex-Jalisco
A-swine-Mexico-AVX-19-2012-H3N2--2012-06-22--Mex-Guanajuato
A-swine-Mexico-AVX-26-2012-H1N2--2012-06-22--Mex-Guanajuato
A-swine-Mexico-AVX-4-2011-H1N2--2011-03-18--Mex-Jalisco
A-swine-Mexico-AVX-16-2012-H1N2--2012-03-04--Mex-Jalisco
A-swine-Mexico-AVX-13-2012-H3N2--2012-01-05--Mex-Jalisco
2000
2005
2010
2015

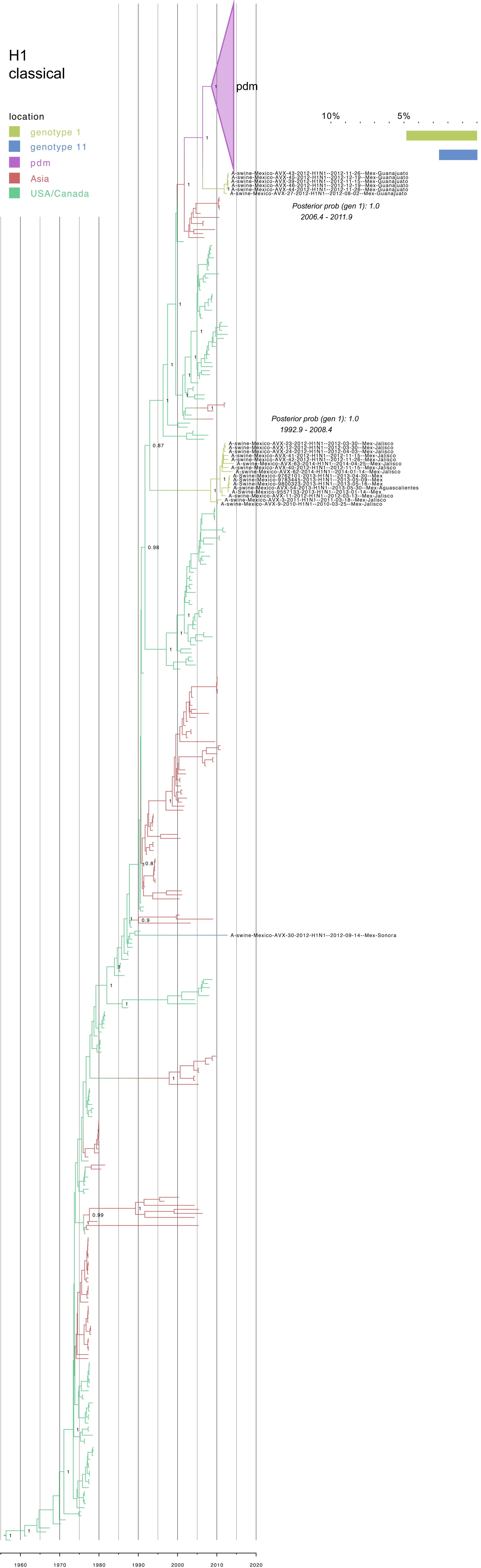

H1
classical
location
genotype 1
genotype 11
pdm
Asia
USA/Canada
pdm
10%
5%
A-swine-Mexico-AVX-43-2012-H1N1--2012-11-26--Mex-Guanajuato
A-swine-Mexico-AVX-45-2012-H1N1--2012-12-19--Mex-Guanajuato
A-swine-Mexico-AVX-39-2012-H1N1--2012-11-15--Mex-Guanajuato
A-swine-Mexico-AVX-46-2012-H1N1--2012-12-19--Mex-Guanajuato
A-swine-Mexico-AVX-44-2012-H1N1--2012-11-26--Mex-Guanajuato
A-swine-Mexico-AVX-27-2012-H1N1--2012-08-02--Mex-Guanajuato
Posterior prob (gen 1): 1.0
2006.4 - 2011.9
Posterior prob (gen 1): 1.0
1992.9 - 2008.4
A-swine-Mexico-AVX-23-2012-H1N1--2012-03-30--Mex-Jalisco
A-swine-Mexico-AVX-12-2012-H1N1--2012-03-30--Mex-Jalisco
A-swine-Mexico-AVX-24-2012-H1N1--2012-04-03--Mex-Jalisco
A-swine-Mexico-AVX-41-2012-H1N1--2012-11-15--Mex-Jalisco
A-swine-Mexico-AVX-42-2012-H1N1--2012-11-26--Mex-Jalisco
A-swine-Mexico-AVX-63-2014-H1N1--2014-04-25--Mex-Jalisco
A-swine-Mexico-AVX-40-2012-H1N1--2012-11-15--Mex-Jalisco
A-swine-Mexico-AVX-62-2014-H1N1--2014-01-14--Mex-Jalisco
A-Swine-Mexico-9762101-2013-H1N1--2013-04-30--Mex
A-Swine-Mexico-9783445-2013-H1N1--2013-05-09--Mex
A-Swine-Mexico-9800323-2013-H1N1--2013-05-16--Mex
A-swine-Mexico-AVX-54-2013-H1N1--2013-05-30--Mex-Aguascalientes
A-Swine-Mexico-9557133-2013-H1N1--2013-01-14--Mex
A-swine-Mexico-AVX-11-2012-H1N1--2012-03-13--Mex-Jalisco
A-swine-Mexico-AVX-3-2011-H1N1--2011-03-18--Mex-Jalisco
A-swine-Mexico-AVX-9-2010-H1N1--2010-03-25--Mex-Jalisco
0.87
0.98
0.8
0.9
A-swine-Mexico-AVX-30-2012-H1N1--2012-09-14--Mex-Sonora
0.99
1960
1970
1980
1990
2000
2010
2020

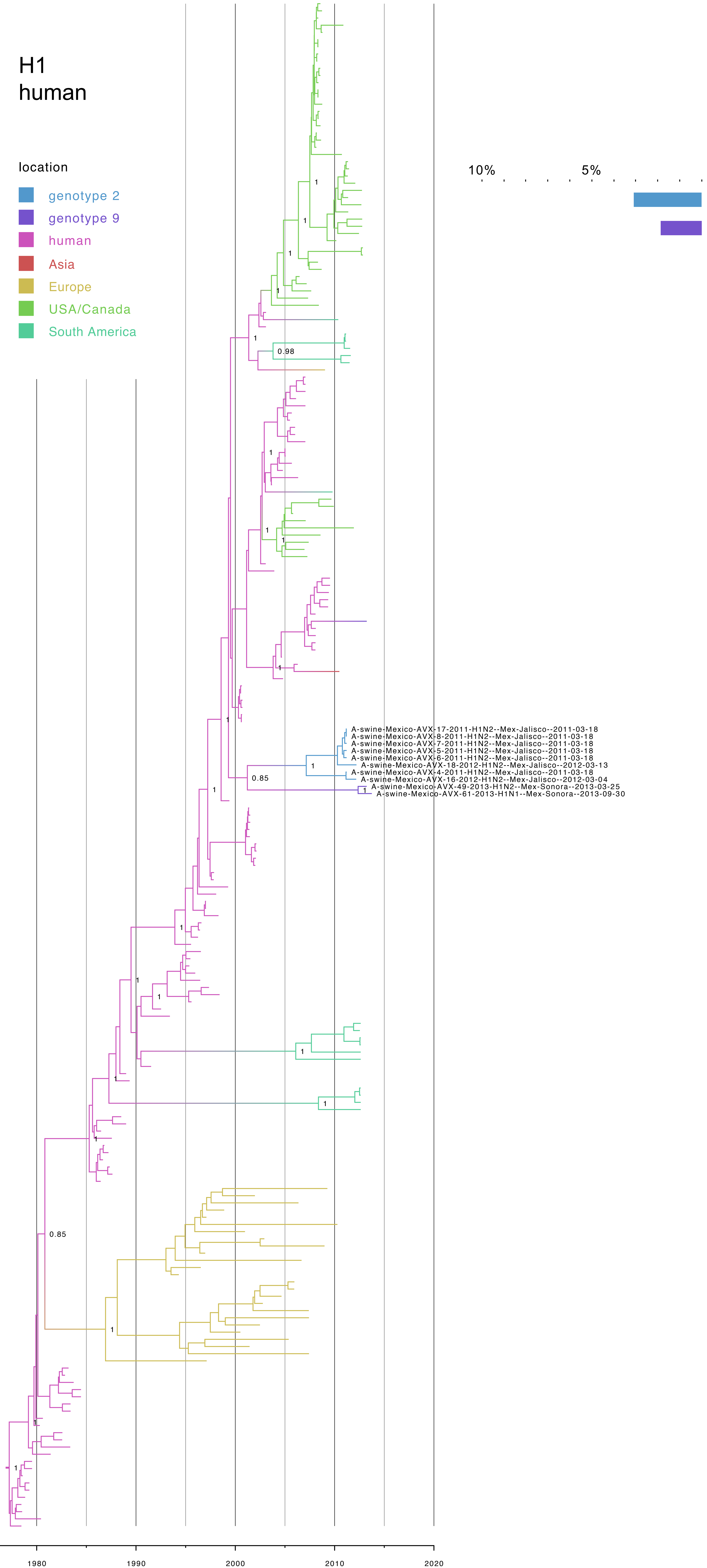

H1
human
location
genotype 2
genotype 9
human
Asia
Europe
USA/Canada
South America
10%
5%
A-swine-Mexico-AVX-17-2011-H1N2--Mex-Jalisco--2011-03-18
A-swine-Mexico-AVX-8-2011-H1N2--Mex-Jalisco--2011-03-18
A-swine-Mexico-AVX-7-2011-H1N2--Mex-Jalisco--2011-03-18
A-swine-Mexico-AVX-5-2011-H1N2--Mex-Jalisco--2011-03-18
A-swine-Mexico-AVX-6-2011-H1N2--Mex-Jalisco--2011-03-18
A-swine-Mexico-AVX-18-2012-H1N2--Mex-Jalisco--2012-03-13
A-swine-Mexico-AVX-4-2011-H1N2--Mex-Jalisco--2011-03-18
A-swine-Mexico-AVX-16-2012-H1N2--Mex-Jalisco--2012-03-04
A-swine-Mexico-AVX-49-2013-H1N2--Mex-Sonora--2013-03-25
A-swine-Mexico-AVX-61-2013-H1N1--Mex-Sonora--2013-09-30
0.98
0.85
0.85
1980
1990
2000
2010
2020

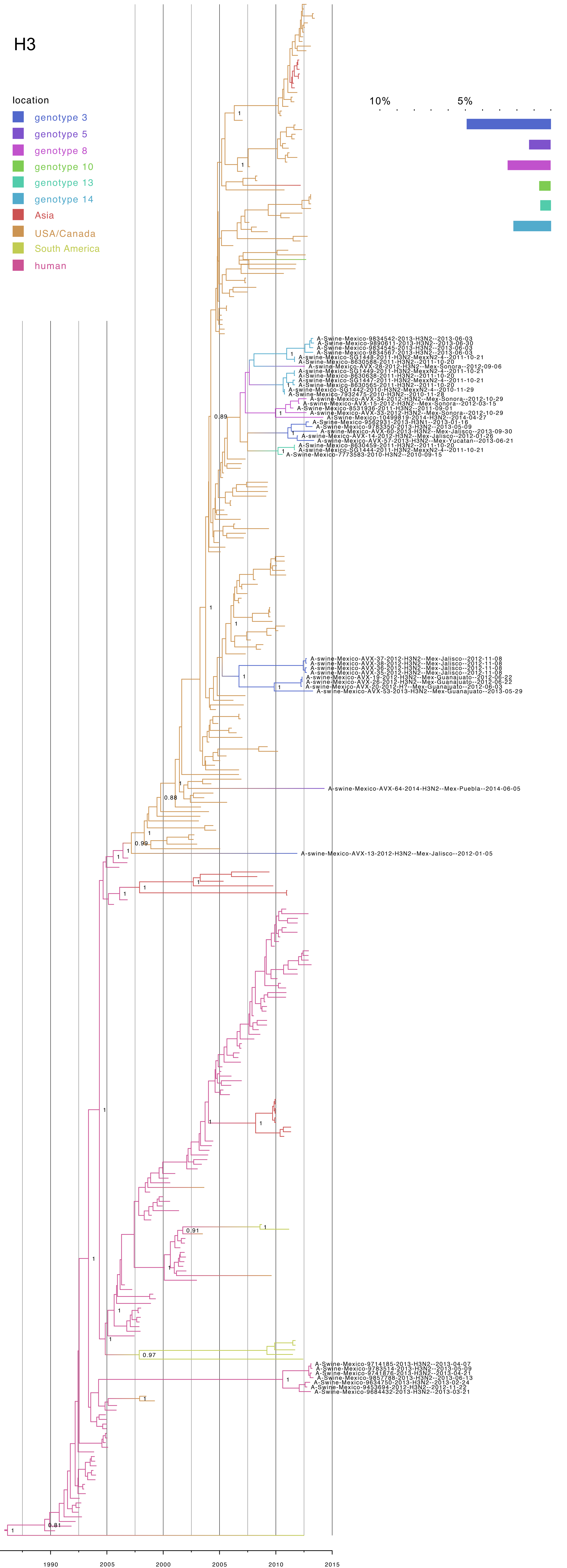
H3
location
genotype 3
genotype 5
genotype 8
genotype 10
genotype 13
genotype 14
Asia
USA/Canada
South America
human
10%
5%
A-Swine-Mexico-9834542-2013-H3N2--2013-06-03
A-Swine-Mexico-9890611-2013-H3N2--2013-06-30
A-Swine-Mexico-9834545-2013-H3N2--2013-06-03
A-Swine-Mexico-9834567-2013-H3N2--2013-06-03
A-swine-Mexico-SG1448-2011-H3N2-MexxN2-4--2011-10-21
A-Swine-Mexico-8630588-2011-H3N2--2011-10-20
A-swine-Mexico-AVX-28-2012-H3N2--Mex-Sonora--2012-09-06
A-swine-Mexico-SG1449-2011-H3N2-MexxN2-4--2011-10-21
A-Swine-Mexico-8630638-2011-H3N2--2011-10-20
A-swine-Mexico-SG1447-2011-H3N2-MexxN2-4--2011-10-21
A-Swine-Mexico-8630565-2011-H3N2--2011-10-20
A-swine-Mexico-SG1442-2010-H3N2-MexxN2-4--2010-11-29
A-Swine-Mexico-7932475-2010-H3N2--2010-11-28
A-swine-Mexico-AVX-34-2012-H3N2--Mex-Sonora--2012-10-29
A-swine-Mexico-AVX-15-2012-H3N2--Mex-Sonora--2012-03-15
A-Swine-Mexico-8531936-2011-H3N2--2011-09-01
A-swine-Mexico-AVX-33-2012-H3N2--Mex-Sonora--2012-10-29
A-Swine-Mexico-10499819-2014-H3N2--2014-04-27
A-Swine-Mexico-9562931-2013-H3N1--2013-01-16
A-Swine-Mexico-9783350-2013-H3N2--2013-05-09
A-swine-Mexico-AVX-60-2013-H3N2--Mex-Jalisco--2013-09-30
A-swine-Mexico-AVX-14-2012-H3N2--Mex-Jalisco--2012-01-26
A-swine-Mexico-AVX-57-2013-H3N2--Mex-Yucatan--2013-06-21
A-Swine-Mexico-8630459-2011-H3N2--2011-10-20
A-swine-Mexico-SG1444-2011-H3N2-MexxN2-4--2011-10-21
A-Swine-Mexico-7773583-2010-H3N2--2010-09-15
A-swine-Mexico-AVX-37-2012-H3N2--Mex-Jalisco--2012-11-08
A-swine-Mexico-AVX-38-2012-H3N2--Mex-Jalisco--2012-11-08
A-swine-Mexico-AVX-36-2012-H3N2--Mex-Jalisco--2012-11-08
A-swine-Mexico-AVX-35-2012-H3N2--Mex-Jalisco--2012-11-08
A-swine-Mexico-AVX-19-2012-H3N2--Mex-Guanajuato--2012-06-22
A-swine-Mexico-AVX-26-2012-H3N2--Mex-Guanajuato--2012-06-22
A-swine-Mexico-AVX-20-2012-H?--Mex-Guanajuato--2012-06-03
A-swine-Mexico-AVX-53-2013-H3N2--Mex-Guanajuato--2013-05-29
A-swine-Mexico-AVX-64-2014-H3N2--Mex-Puebla--2014-06-05
A-swine-Mexico-AVX-13-2012-H3N2--Mex-Jalisco--2012-01-05
A-Swine-Mexico-9714185-2013-H3N2--2013-04-07
A-Swine-Mexico-9783514-2013-H3N2--2013-05-09
A-Swine-Mexico-9741876-2013-H3N2--2013-04-21
A-Swine-Mexico-9857788-2013-H3N2--2013-06-13
A-Swine-Mexico-9634750-2013-H3N2--2013-02-24
A-Swine-Mexico-9453694-2012-H3N2--2012-11-22
A-Swine-Mexico-9684432-2013-H3N2--2013-03-21
1990
2005
2000
2005
2010
2015

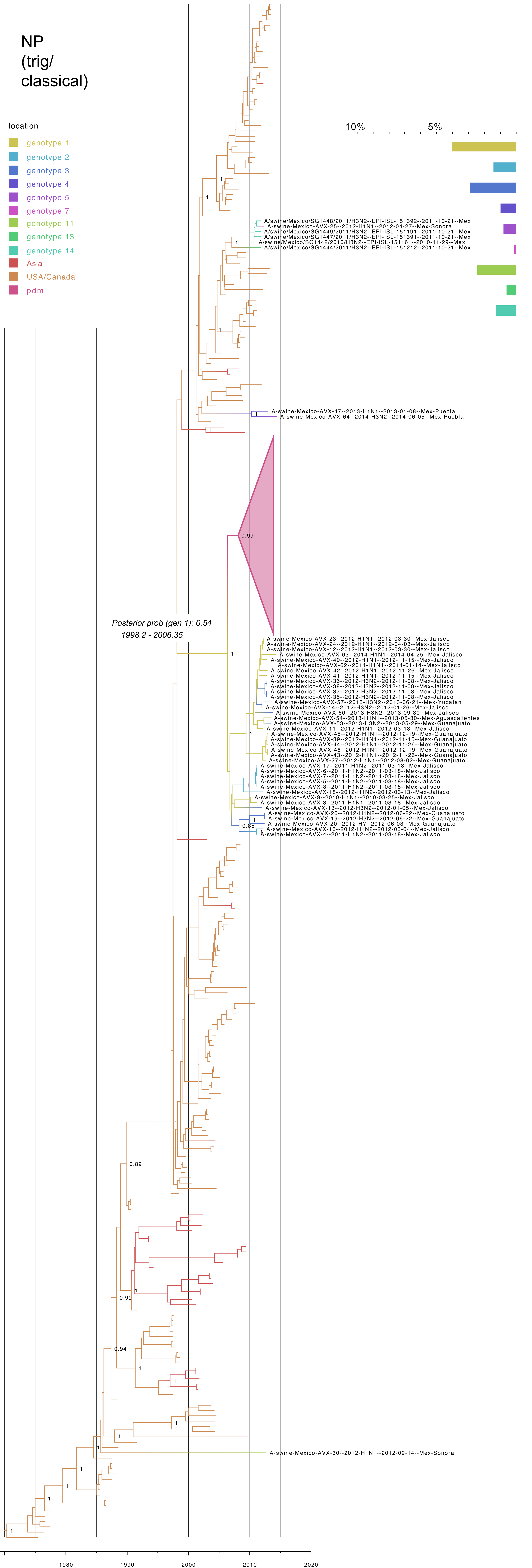

NP
(trig/
classical)
location
genotype 1
genotype 2
genotype 3
genotype 4
genotype 5
genotype 7
genotype 11
genotype 13
genotype 14
Asia
USA/Canada
pdm
10%
5%
A/swine/Mexico/SG1448/2011/H3N2--EPI-ISL-151392--2011-10-21--Mex
A-swine-Mexico-AVX-25--2012-H1N1--2012-04-27--Mex-Sonora
A/swine/Mexico/SG1449/2011/H3N2--EPI-ISL-151191--2011-10-21--Mex
A/swine/Mexico/SG1447/2011/H3N2--EPI-ISL-151391--2011-10-21--Mex
A/swine/Mexico/SG1442/2010/H3N2--EPI-ISL-151161--2010-11-29--Mex
A/swine/Mexico/SG1444/2011/H3N2--EPI-ISL-151212--2011-10-21--Mex
A-swine-Mexico-AVX-47--2013-H1N1--2013-01-08--Mex-Puebla
A-swine-Mexico-AVX-64--2014-H3N2--2014-06-05--Mex-Puebla
Posterior prob (gen 1): 0.54
1998.2 - 2006.35
A-swine-Mexico-AVX-23--2012-H1N1--2012-03-30--Mex-Jalisco
A-swine-Mexico-AVX-24--2012-H1N1--2012-04-03--Mex-Jalisco
A-swine-Mexico-AVX-12--2012-H1N1--2012-03-30--Mex-Jalisco
A-swine-Mexico-AVX-63--2014-H1N1--2014-04-25--Mex-Jalisco
A-swine-Mexico-AVX-40--2012-H1N1--2012-11-15--Mex-Jalisco
A-swine-Mexico-AVX-62--2014-H1N1--2014-01-14--Mex-Jalisco
A-swine-Mexico-AVX-42--2012-H1N1--2012-11-26--Mex-Jalisco
A-swine-Mexico-AVX-41--2012-H1N1--2012-11-15--Mex-Jalisco
A-swine-Mexico-AVX-36--2012-H3N2--2012-11-08--Mex-Jalisco
A-swine-Mexico-AVX-38--2012-H3N2--2012-11-08--Mex-Jalisco
A-swine-Mexico-AVX-37--2012-H3N2--2012-11-08--Mex-Jalisco
A-swine-Mexico-AVX-35--2012-H3N2--2012-11-08--Mex-Jalisco
A-swine-Mexico-AVX-57--2013-H3N2--2013-06-21--Mex-Yucatan
A-swine-Mexico-AVX-14--2012-H3N2--2012-01-26--Mex-Jalisco
A-swine-Mexico-AVX-60--2013-H3N2--2013-09-30--Mex-Jalisco
A-swine-Mexico-AVX-54--2013-H1N1--2013-05-30--Mex-Aguascalientes
A-swine-Mexico-AVX-53--2013-H3N2--2013-05-29--Mex-Guanajuato
A-swine-Mexico-AVX-11--2012-H1N1--2012-03-13--Mex-Jalisco
A-swine-Mexico-AVX-45--2012-H1N1--2012-12-19--Mex-Guanajuato
A-swine-Mexico-AVX-39--2012-H1N1--2012-11-15--Mex-Guanajuato
A-swine-Mexico-AVX-44--2012-H1N1--2012-11-26--Mex-Guanajuato
A-swine-Mexico-AVX-46--2012-H1N1--2012-12-19--Mex-Guanajuato
A-swine-Mexico-AVX-43--2012-H1N1--2012-11-26--Mex-Guanajuato
A-swine-Mexico-AVX-27--2012-H1N1--2012-08-02--Mex-Guanajuato
A-swine-Mexico-AVX-17--2011-H1N2--2011-03-18--Mex-Jalisco
A-swine-Mexico-AVX-6--2011-H1N2--2011-03-18--Mex-Jalisco
A-swine-Mexico-AVX-7--2011-H1N2--2011-03-18--Mex-Jalisco
A-swine-Mexico-AVX-5--2011-H1N2--2011-03-18--Mex-Jalisco
A-swine-Mexico-AVX-8--2011-H1N2--2011-03-18--Mex-Jalisco
A-swine-Mexico-AVX-18--2012-H1N2--2012-03-13--Mex-Jalisco
A-swine-Mexico-AVX-9--2010-H1N1--2010-03-25--Mex-Jalisco
A-swine-Mexico-AVX-3--2011-H1N1--2011-03-18--Mex-Jalisco
A-swine-Mexico-AVX-13--2012-H3N2--2012-01-05--Mex-Jalisco
A-swine-Mexico-AVX-26--2012-H1N2--2012-06-22--Mex-Guanajuato
A-swine-Mexico-AVX-19--2012-H3N2--2012-06-22--Mex-Guanajuato
A-swine-Mexico-AVX-20--2012-H?--2012-06-03--Mex-Guanajuato
A-swine-Mexico-AVX-16--2012-H1N2--2012-03-04--Mex-Jalisco
A-swine-Mexico-AVX-4--2011-H1N2--2011-03-18--Mex-Jalisco
A-swine-Mexico-AVX-30--2012-H1N1--2012-09-14--Mex-Sonora
1980
1990
2000
2010
2020

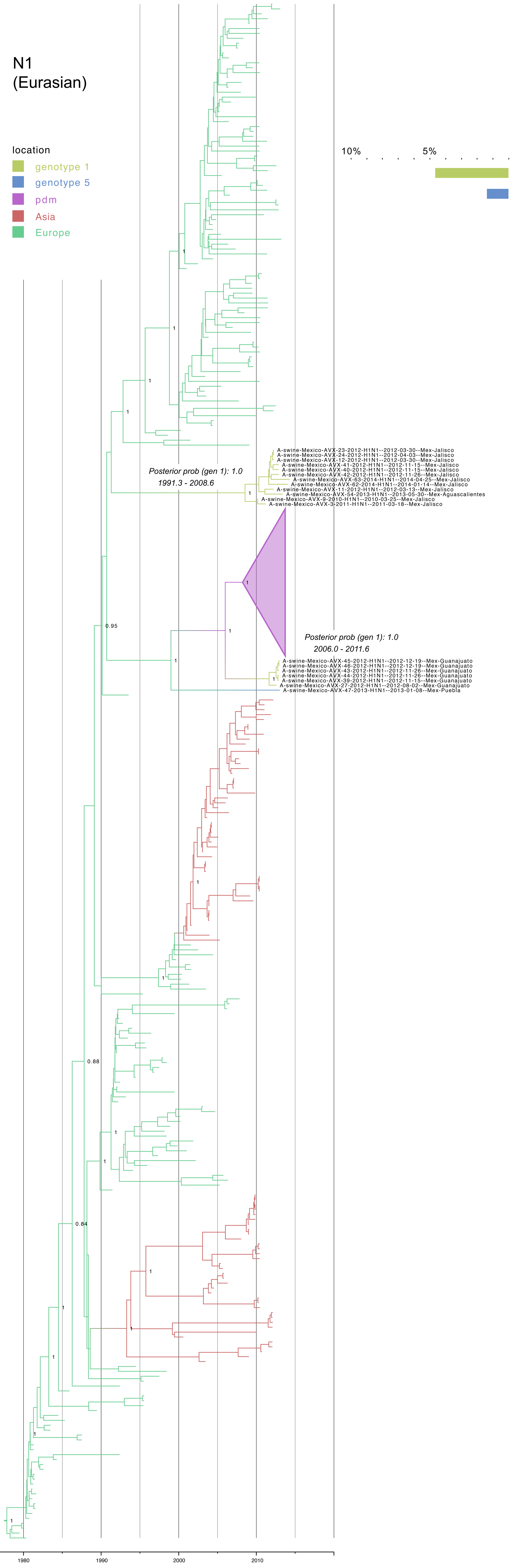
N1
(Eurasian)
location
genotype 1
genotype 5
pdm
Asia
Europe
10%
5%
Posterior prob (gen 1): 1.0
1991.3 - 2008.6
A-swine-Mexico-AVX-23-2012-H1N1--2012-03-30--Mex-Jalisco
A-swine-Mexico-AVX-24-2012-H1N1--2012-04-03--Mex-Jalisco
A-swine-Mexico-AVX-12-2012-H1N1--2012-03-30--Mex-Jalisco
A-swine-Mexico-AVX-41-2012-H1N1--2012-11-15--Mex-Jalisco
A-swine-Mexico-AVX-40-2012-H1N1--2012-11-15--Mex-Jalisco
A-swine-Mexico-AVX-42-2012-H1N1--2012-11-26--Mex-Jalisco
A-swine-Mexico-AVX-63-2014-H1N1--2014-04-25--Mex-Jalisco
A-swine-Mexico-AVX-62-2014-H1N1--2014-01-14--Mex-Jalisco
A-swine-Mexico-AVX-11-2012-H1N1--2012-03-13--Mex-Jalisco
A-swine-Mexico-AVX-54-2013-H1N1--2013-05-30--Mex-Aguascalientes
A-swine-Mexico-AVX-9-2010-H1N1--2010-03-25--Mex-Jalisco
A-swine-Mexico-AVX-3-2011-H1N1--2011-03-18--Mex-Jalisco
Posterior prob (gen 1): 1.0
2006.0 - 2011.6
A-swine-Mexico-AVX-45-2012-H1N1--2012-12-19--Mex-Guanajuato
A-swine-Mexico-AVX-46-2012-H1N1--2012-12-19--Mex-Guanajuato
A-swine-Mexico-AVX-43-2012-H1N1--2012-11-26--Mex-Guanajuato
A-swine-Mexico-AVX-44-2012-H1N1--2012-11-26--Mex-Guanajuato
A-swine-Mexico-AVX-39-2012-H1N1--2012-11-15--Mex-Guanajuato
A-swine-Mexico-AVX-27-2012-H1N1--2012-08-02--Mex-Guanajuato
A-swine-Mexico-AVX-47-2013-H1N1--2013-01-08--Mex-Puebla
0.95
0.88
0.84
1980
1990
2000
2010

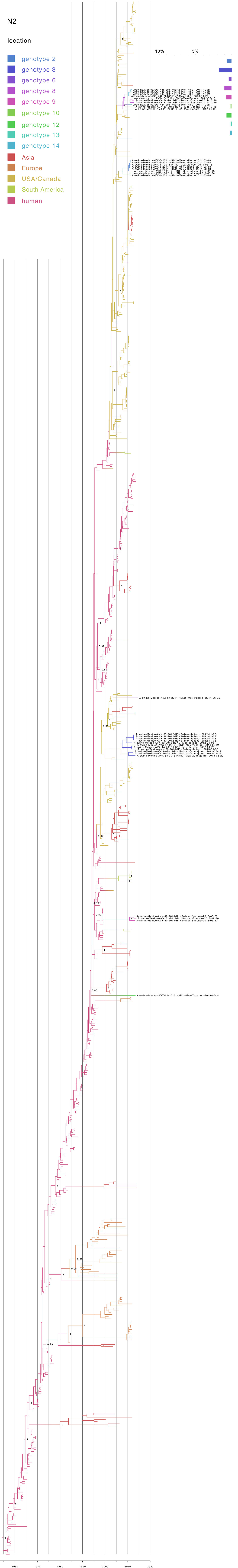

N2
location
genotype 2
genotype 3
genotype 6
genotype 8
genotype 9
genotype 10
genotype 12
genotype 13
genotype 14
Asia
Europe
USA/Canada
South America
human
10%
5%
A/swine/Mexico/SG1448/2011/H3N2-Mex-H3-3--2011-10-21
A/swine/Mexico/SG1449/2011/H3N2-Mex-H3-3--2011-10-21
A/swine/Mexico/SG1447/2011/H3N2-Mex-H3-3--2011-10-21
A/swine/Mexico/SG1442/2010/H3N2-Mex-H3-3--2010-11-29
A-swine-Mexico-AVX-15-2012-H3N2--Mex-Sonora--2012-03-15
A-swine-Mexico-AVX-34-2012-H3N2--Mex-Sonora--2012-10-29
A-swine-Mexico-AVX-33-2012-H3N2--Mex-Sonora--2012-10-29
A/swine/Mexico/SG1444/2011/H3N2-Mex-H3-3--2011-10-21
A-swine-Mexico-AVX-32-2012-H3N2--Mex-Sonora--2012-10-18
A-swine-Mexico-AVX-28-2012-H3N2--Mex-Sonora--2012-09-06
A-swine-Mexico-AVX-6-2011-H1N2--Mex-Jalisco--2011-03-18
A-swine-Mexico-AVX-8-2011-H1N2--Mex-Jalisco--2011-03-18
A-swine-Mexico-AVX-17-2011-H1N2--Mex-Jalisco--2011-03-18
A-swine-Mexico-AVX-5-2011-H1N2--Mex-Jalisco--2011-03-18
A-swine-Mexico-AVX-7-2011-H1N2--Mex-Jalisco--2011-03-18
A-swine-Mexico-AVX-18-2012-H1N2--Mex-Jalisco--2012-03-13
A-swine-Mexico-AVX-16-2012-H1N2--Mex-Jalisco--2012-03-04
A-swine-Mexico-AVX-4-2011-H1N2--Mex-Jalisco--2011-03-18
A-swine-Mexico-AVX-64-2014-H3N2--Mex-Puebla--2014-06-05
A-swine-Mexico-AVX-35-2012-H3N2--Mex-Jalisco--2012-11-08
A-swine-Mexico-AVX-36-2012-H3N2--Mex-Jalisco--2012-11-08
A-swine-Mexico-AVX-38-2012-H3N2--Mex-Jalisco--2012-11-08
A-swine-Mexico-AVX-37-2012-H3N2--Mex-Jalisco--2012-11-08
A-swine-Mexico-AVX-13-2012-H3N2--Mex-Jalisco--2012-01-05
A-swine-Mexico-AVX-57-2013-H3N2--Mex-Yucatan--2013-06-21
A-swine-Mexico-AVX-14-2012-H3N2--Mex-Jalisco--2012-01-26
A-swine-Mexico-AVX-60-2013-H3N2--Mex-Jalisco--2013-09-30
A-swine-Mexico-AVX-19-2012-H3N2--Mex-Guanajuato--2012-06-22
A-swine-Mexico-AVX-26-2012-H1N2--Mex-Guanajuato--2012-06-22
A-swine-Mexico-AVX-53-2013-H3N2--Mex-Guanajuato--2013-05-29
A-swine-Mexico-AVX-49-2013-H1N2--Mex-Sonora--2013-03-25
A-swine-Mexico-AVX-61-2013-H1N1--Mex-Sonora--2013-09-30
A-swine-Mexico-AVX-50-2013-H1N2--Mex-Sonora--2013-03-27
A-swine-Mexico-AVX-55-2013-H1N2--Mex-Yucatan--2013-06-21
1960
1970
1980
1990
2000
2010
2020

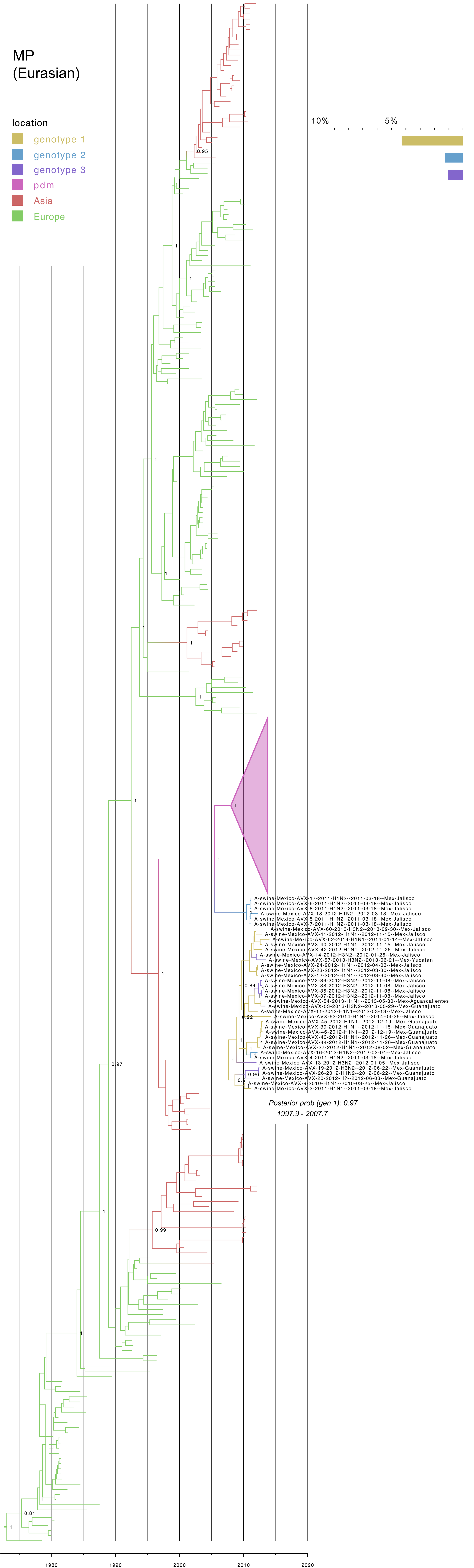
MP
(Eurasian)
location
genotype 1
genotype 2
genotype 3
pdm
Asia
Europe
10%
5%
A-swine-Mexico-AVX-17-2011-H1N2--2011-03-18--Mex-Jalisco
A-swine-Mexico-AVX-6-2011-H1N2--2011-03-18--Mex-Jalisco
A-swine-Mexico-AVX-8-2011-H1N2--2011-03-18--Mex-Jalisco
A-swine-Mexico-AVX-18-2012-H1N2--2012-03-13--Mex-Jalisco
A-swine-Mexico-AVX-5-2011-H1N2--2011-03-18--Mex-Jalisco
A-swine-Mexico-AVX-7-2011-H1N2--2011-03-18--Mex-Jalisco
A-swine-Mexico-AVX-60-2013-H3N2--2013-09-30--Mex-Jalisco
A-swine-Mexico-AVX-41-2012-H1N1--2012-11-15--Mex-Jalisco
A-swine-Mexico-AVX-62-2014-H1N1--2014-01-14--Mex-Jalisco
A-swine-Mexico-AVX-40-2012-H1N1--2012-11-15--Mex-Jalisco
A-swine-Mexico-AVX-42-2012-H1N1--2012-11-26--Mex-Jalisco
A-swine-Mexico-AVX-14-2012-H3N2--2012-01-26--Mex-Jalisco
A-swine-Mexico-AVX-57-2013-H3N2--2013-06-21--Mex-Yucatan
A-swine-Mexico-AVX-24-2012-H1N1--2012-04-03--Mex-Jalisco
A-swine-Mexico-AVX-23-2012-H1N1--2012-03-30--Mex-Jalisco
A-swine-Mexico-AVX-12-2012-H1N1--2012-03-30--Mex-Jalisco
A-swine-Mexico-AVX-36-2012-H3N2--2012-11-08--Mex-Jalisco
A-swine-Mexico-AVX-38-2012-H3N2--2012-11-08--Mex-Jalisco
A-swine-Mexico-AVX-35-2012-H3N2--2012-11-08--Mex-Jalisco
A-swine-Mexico-AVX-37-2012-H3N2--2012-11-08--Mex-Jalisco
A-swine-Mexico-AVX-54-2013-H1N1--2013-05-30--Mex-Aguascalientes
A-swine-Mexico-AVX-53-2013-H3N2--2013-05-29--Mex-Guanajuato
A-swine-Mexico-AVX-11-2012-H1N1--2012-03-13--Mex-Jalisco
A-swine-Mexico-AVX-63-2014-H1N1--2014-04-25--Mex-Jalisco
A-swine-Mexico-AVX-45-2012-H1N1--2012-12-19--Mex-Guanajuato
A-swine-Mexico-AVX-39-2012-H1N1--2012-11-15--Mex-Guanajuato
A-swine-Mexico-AVX-46-2012-H1N1--2012-12-19--Mex-Guanajuato
A-swine-Mexico-AVX-43-2012-H1N1--2012-11-26--Mex-Guanajuato
A-swine-Mexico-AVX-44-2012-H1N1--2012-11-26--Mex-Guanajuato
A-swine-Mexico-AVX-27-2012-H1N1--2012-08-02--Mex-Guanajuato
A-swine-Mexico-AVX-16-2012-H1N2--2012-03-04--Mex-Jalisco
A-swine-Mexico-AVX-4-2011-H1N2--2011-03-18--Mex-Jalisco
A-swine-Mexico-AVX-13-2012-H3N2--2012-01-05--Mex-Jalisco
A-swine-Mexico-AVX-19-2012-H3N2--2012-06-22--Mex-Guanajuato
A-swine-Mexico-AVX-26-2012-H1N2--2012-06-22--Mex-Guanajuato
A-swine-Mexico-AVX-20-2012-H?--2012-06-03--Mex-Guanajuato
A-swine-Mexico-AVX-9-2010-H1N1--2010-03-25--Mex-Jalisco
A-swine-Mexico-AVX-3-2011-H1N1--2011-03-18--Mex-Jalisco
Posterior prob (gen 1): 0.97
1997.9 - 2007.7
1980
1990
2000
2010
2020

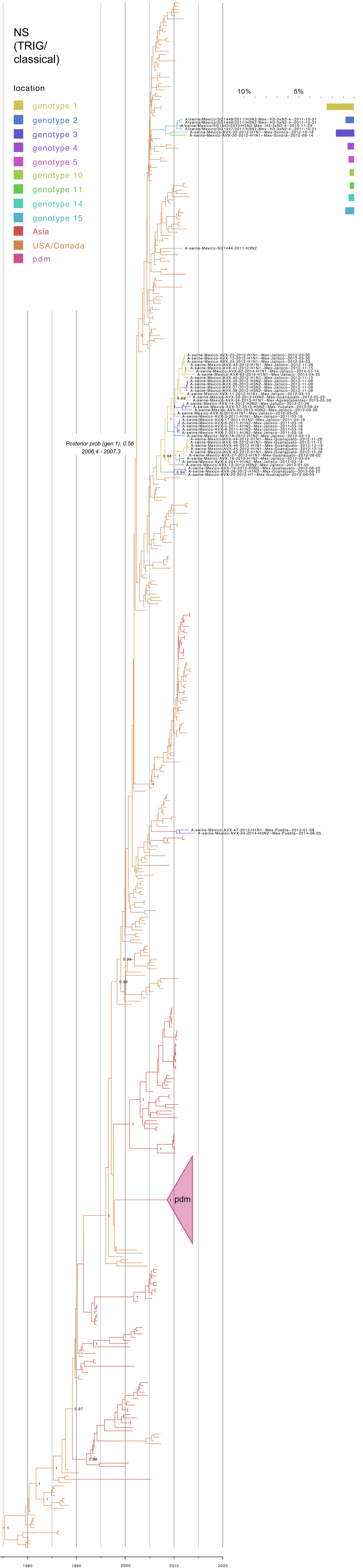
NS
(TRIG/
classical)
location
genotype 1
genotype 2
genotype 3
genotype 4
genotype 5
genotype 10
genotype 11
genotype 14
genotype 15
Asia
USA/Canada
pdm
10%
5%
A/swine/Mexico/SG1448/2011/H3N2-Mex--H3-3xN2-4--2011-10-21
A/swine/Mexico/SG1449/2011/H3N2-Mex--H3-3xN2-4--2011-10-21
A/swine/Mexico/SG1442/2010/H3N2-Mex--H3-3xN2-4--2010-11-29
A/swine/Mexico/SG1447/2011/H3N2-Mex--H3-3xN2-4--2011-10-21
A-swine-Mexico-AVX-32-2012-H1N1--Mex-Sonora--2012-10-18
A-swine-Mexico-AVX-30-2012-H1N1--Mex-Sonora--2012-09-14
A-swine-Mexico-SG1444-2011-H3N2
A-swine-Mexico-AVX-23-2012-H1N1--Mex-Jalisco--2012-03-30
A-swine-Mexico-AVX-12-2012-H1N1--Mex-Jalisco--2012-03-30
A-swine-Mexico-AVX-24-2012-H1N1--Mex-Jalisco--2012-04-03
A-swine-Mexico-AVX-42-2012-H1N1--Mex-Jalisco--2012-11-26
A-swine-Mexico-AVX-41-2012-H1N1--Mex-Jalisco--2012-11-15
A-swine-Mexico-AVX-62-2014-H1N1--Mex-Jalisco--2014-01-14
A-swine-Mexico-AVX-63-2014-H1N1--Mex-Jalisco--2014-04-25
A-swine-Mexico-AVX-40-2012-H1N1--Mex-Jalisco--2012-11-15
A-swine-Mexico-AVX-35-2012-H3N2--Mex-Jalisco--2012-11-08
A-swine-Mexico-AVX-36-2012-H3N2--Mex-Jalisco--2012-11-08
A-swine-Mexico-AVX-37-2012-H3N2--Mex-Jalisco--2012-11-08
A-swine-Mexico-AVX-38-2012-H3N2--Mex-Jalisco--2012-11-08
A-swine-Mexico-AVX-11-2012-H1N1--Mex-Jalisco--2012-03-13
A-swine-Mexico-AVX-53-2013-H3N2--Mex-Guanajuato--2013-05-29
A-swine-Mexico-AVX-54-2013-H1N1--Mex-Aguascalientes--2013-05-30
A-swine-Mexico-AVX-14-2012-H3N2--Mex-Jalisco--2012-01-26
A-swine-Mexico-AVX-57-2013-H3N2--Mex-Yucatan--2013-06-21
A-swine-Mexico-AVX-60-2013-H3N2--Mex-Jalisco--2013-09-30
A-swine-Mexico-AVX-9-2010-H1N1--Mex-Jalisco--2010-03-25
A-swine-Mexico-AVX-3-2011-H1N1--Mex-Jalisco--2011-03-18
A-swine-Mexico-AVX-17-2011-H1N2--Mex-Jalisco--2011-03-18
A-swine-Mexico-AVX-6-2011-H1N2--Mex-Jalisco--2011-03-18
A-swine-Mexico-AVX-5-2011-H1N2--Mex-Jalisco--2011-03-18
A-swine-Mexico-AVX-8-2011-H1N2--Mex-Jalisco--2011-03-18
A-swine-Mexico-AVX-7-2011-H1N2--Mex-Jalisco--2011-03-18
A-swine-Mexico-AVX-18-2012-H1N2--Mex-Jalisco--2012-03-13
A-swine-Mexico-AVX-44-2012-H1N1--Mex-Guanajuato--2012-11-26
A-swine-Mexico-AVX-39-2012-H1N1--Mex-Guanajuato--2012-11-15
A-swine-Mexico-AVX-46-2012-H1N1--Mex-Guanajuato--2012-12-19
A-swine-Mexico-AVX-45-2012-H1N1--Mex-Guanajuato--2012-12-19
A-swine-Mexico-AVX-43-2012-H1N1--Mex-Guanajuato--2012-11-26
A-swine-Mexico-AVX-27-2012-H1N1--Mex-Guanajuato--2012-08-02
A-swine-Mexico-AVX-16-2012-H1N2--Mex-Jalisco--2012-03-04
A-swine-Mexico-AVX-4-2011-H1N2--Mex-Jalisco--2011-03-18
A-swine-Mexico-AVX-13-2012-H3N2--Mex-Jalisco--2012-01-05
A-swine-Mexico-AVX-19-2012-H3N2--Mex-Guanajuato--2012-06-22
A-swine-Mexico-AVX-26-2012-H1N2--Mex-Guanajuato--2012-06-22
A-swine-Mexico-AVX-20-2012-H?--Mex-Guanajuato--2012-06-03
Posterior prob (gen 1): 0.56
2006.4 - 2007.3
A-swine-Mexico-AVX-47-2013-H1N1--Mex-Puebla--2013-01-08
A-swine-Mexico-AVX-64-2014-H3N2--Mex-Puebla--2014-06-05
pdm
1980
1990
2000
2010
2020
