# Supplementary figures and images for: Origins of the 2009 H1N1 influenza pandemic in swine in Mexico
Source: eLife. 2016 Jun 28;5:e16777. doi: 10.7554/eLife.16777 (PMC4957980; doi:10.7554/eLife.16777)

PB2  
(pdmH1N1)

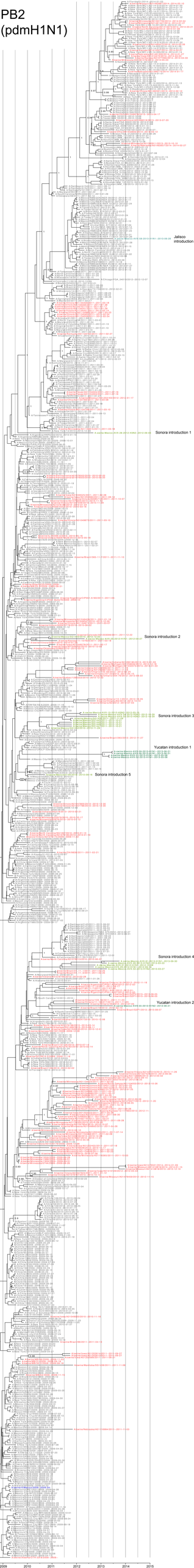

Supplement: Figure 3—source data 4. — Evolutionary relationships of pdmH1N1 viruses collected from humans and swine, 2009–2014 for the representative PB2 and HA segments. Separate viral introductions from humans into Mexican swine are indicated. DOI: http://dx.doi.org/10.7554/eLife.16777.011 [file elife-16777-fig3-data4.pdf]
