# Supplementary material for: Origins of the 2009 H1N1 influenza pandemic in swine in Mexico
Source: eLife. 2016 Jun 28;5:e16777. doi: 10.7554/eLife.16777 (PMC4957980; doi:10.7554/eLife.16777)
Supplement: Reporting Standard 1. — DOI: http://dx.doi.org/10.7554/eLife.16777.028 [file elife-16777-supp1.pdf]

DPCC FASTA Reference for Sequence Submission v2.0

	Sequence_Identifier	Sample_Identifier	Segment	Clone_Identifier
**Input Type**	Text Field	Text Field	Text Field	Text Field
**Definition**	A unique identifier for each sequence in a submission	The identifier assigned to the virus strain	The viral gene name of the sequence submission	The identifier assigned to the individual clone of the influenza virus genome segment
**Format**	Text<br><br>Maximum length: 90 characters	Text<br><br>Maximum length: 50 characters	Text<br><br>Maximum length: 25 characters	Text<br><br>Maximum length: 15 characters
**Value List**	None	None	Influenza A virus:<br>PB2, PB1, PA, HA, NP, NA, MP, or NS<br><br>Influenza B virus:<br>PB1, PB2, PA, HA, NP, NA, MP, or NS<br><br>Influenza C virus:<br>PB2, PB1, P3, HE, NP, MP, or NS<br><br>Influenza D virus:<br>PB2, PB1, P3, HE, NP, P42, or NS<br><br>Other virus types:<br>Enter gene name	None
**Curation**	The entry must be unique for each sequence in the FASTA file. Data elements must match those listed in the Sample_Identifer, Segment, and optional Clone_Identifier fields.	The Sample_Identifier initially assigned to the surveillance sample must be provided.	No spaces are allowed.<br><br>Segment must match the influenza virus type (A, B, C, or D) listed in the Strain_Name field on the Sequence Metadata template.<br><br>For other virus types, if the sequence covers multiple genes, comma-separate individual gene names.	No spaces are allowed.<br><br>Optional field
**Examples**	22258468.PB2 or<br>452134.HA.1 or<br>452134.HA.2 or<br>452134.NA.Major_variant or<br>452134.NA.Minor_variant	22258468 or<br>452134	PB2 or<br>HA or<br>RdRp or<br>Capsid,RdRp	1 or<br>2 or<br>Major_variant or<br>Minor_variant
**Notes**		Submissions without a corresponding surveillance submission will be listed as orphaned records.<br><br>For non-surveillance submissions, enter a unique Sample_Identifier.		This field is not required when submitting a single sequence for an individual genome Segment.<br><br>This field is required when submitting multiple sequences for the same Segment and Sample_Identifier i.e., from mixed or co-infected samples.
