# Supplementary material for: Origins of the 2009 H1N1 influenza pandemic in swine in Mexico
Source: eLife. 2016 Jun 28;5:e16777. doi: 10.7554/eLife.16777 (PMC4957980; doi:10.7554/eLife.16777)
Supplement: Reporting Standard 2. — DOI: http://dx.doi.org/10.7554/eLife.16777.029 [file elife-16777-supp2.pdf]

DPCC METADATA Reference for Sequence Submission v2.0

Global validation rules:

1. All fields are required and cannot be blank
2. The capital letter U for "Unknown" can be used in place of blank
3. The abbreviation NA is used for "Not Available" or "Not Applicable"
4. The OTH- abbreviation is always used for "Other"
5. Use comma (,) to separate multiple values in the same field
6. Use double quotes (" ") to escape entries with commas when not intended to separate multiple values (e.g., "Jones, Indiana")
7. Controlled values are case-sensitive and must be entered in all-caps

	Project_Identifier	Contributing_Institution	Sample_Identifier	BioProject_Accession_Number	Embargo_End_Date	Provisional_Authors	Submission_Title
**Input Type**	Text Field	Text Field	Text Field	Text Field	Date Field	Text Field	Text Field
**Definition**	A unique Project Identifier generated by the DPCC by combining the Center-generated Project Code and a random 4-digit number	The institution code for the group that PERFORMED THE ANALYSIS. Must be one of the CEIRS institution codes assigned by NIAID	Identifier initially assigned to each sample collected. If multiple samples are taken from the same host, each sample should have its own identifier.	The BioProject unique accession number associated with the sequence submission	In case an embargo of the information is needed, the date that the information should be released to the public databases by the DPCC	The list of authors associated with the sequence submission. Default entry in absence of a Publication_PMID will be the authors listed by the user. If a Publication_PMID is provided, publication authors will be listed.	The descriptive title assigned to the sequence submission. Default entry in absence of a Publication_PMID will be "Direct Submission (CEIRS DPCC)". If a Publication_PMID is provided, Submission_Title should be the title of the referenced publication.
**Format**	Project_Code_XXXX<br><br>Maximum length: 21 characters	Center three-letter code followed by three digits.<br><br>Maximum length: 6 characters	Center-specific<br><br>Maximum length: 50 characters	BioProject ID<br><br>Maximum length: 15 characters	DD-Mon-YYYY<br>DD-Mon-YY<br>NA<br><br>Maximum length: 11 characters	FirstName LastName<br>FirstName MI. LastName<br><br>Maximum length: 50 characters	Text<br><br>Maximum length: 100 characters
**Value List**	None	None	None	None	Date<br>NA	Text<br>NA	Text<br>NA
**Curation**	The entry must be a Project Identifier value registered with the DPCC.	The entry must be an Institution Code value registered with the DPCC.<br><br>Center 3-letter codes are case-sensitive and must be entered in all-caps.	The Sample_Identifier initially assigned to the surveillance sample must be provided.	The entry must be a valid BioProject accession number previously registered with the DPCC.	1. Leading 0 in DD is optional.<br>2. Month must match the first three letters of the month. Month is NOT case-sensitive.<br>3. Years may have two or four digits.<br>4. Date must conform to NIAID data release policies.	If a Publication_PMID is entered, use NA. The list of authors from the publication will be used.	If no Publication_PMID is provided, use NA.<br><br>If a Publication_PMID is entered, use the title of the publication exactly.
**Examples**	SJCProj02_4001	SJC101	22258468	PRJNA37813	3-Mar-2011 or 03-Mar-2011 or 03-MAR-2011 or 3-MAR-11 or NA	Indiana Jones, John M. Henry or NA	Analysis of pandemic influenza H1H1 sequences from New York in 2010 or NA
**Notes**			Submissions without a corresponding surveillance submission will be listed as orphaned records.<br><br>For non-surveillance submissions, enter a unique Sample_Identifier.	The sequence must be linked to a BioProject.<br><br>If no relevant BioProject exists, one must be created.	An exact date must be provided.<br><br>If Embargo_End_Date is NA, information will be released without delay.<br><br>Embargo_End_Date cannot be more than 12 months after submission.	The DPCC will update the GenBank entry records if and when the authors provide a publication reference after submission.<br><br>For listing of multiple names, comma-separate the names maintaining the order of FirstName and LastName.	The DPCC will update the GenBank entry records if and when the authors provide a publication reference after submission.

	Publication_PMID	Molecule_Type	Sequencing_Technology	Forward_Primer	Reverse_Primer	Assembly_Method	Assembler_Version	Coverage
**Input Type**	Text Field	Text Field	Text Field	Text Field	Text Field	Text Field	Text Field	Text Field
**Definition**	The PubMed Unique Identifier (PMID) for the publication in which the sequence was published	The type of organic molecule that was sequenced	The name of the sequencing technology used to obtain the submitted sequences	The forward PCR primer that was used to amplify the nucleic acid that was sequenced	The reverse PCR primer that was used to amplify the nucleic acid that was sequenced	The name of the program used to assemble next-generation sequence reads	The version of the assembly program used or, if not available, the date the assemblies were made	The average number of reads representing a given nucleotide in the sequence
**Format**	PMID<br><br>Maximum length: 50 characters	Text<br><br>Maximum length: 15 characters	Text<br><br>Maximum length: 150 characters	Name:Sequence<br><br>Maximum length: 50 characters	Name:Sequence<br><br>Maximum length: 50 characters	Text<br><br>Maximum length: 150 characters	Text<br>DD-MON-YYYY<br>DD-MON-YY<br>D-MON-YYYY<br>D-MON-YY<br><br>Maximum length: 50 characters	Text<br><br>Maximum length: 50 characters
**Value List**	Text<br>NA	genomic DNA<br>genomic RNA<br>mRNA<br>other RNA<br>other DNA<br>rRNA<br>transcribed RNA<br>tRNA<br>unassigned DNA<br>unassigned RNA<br>viral cRNA	454<br>Helicos<br>Illumina<br>IonTorrent<br>Pacific Biosciences<br>Sanger<br>SOLiD<br>OTH-	Text<br>U	Text<br>U	None	None	Number<br>U
**Curation**	The entry must be a valid PMID number: 7 or 8-digit number with no leading zeros.<br>http://www.nlm.nih.gov/bsd/mms/medlineelements.html#pmid	The entry must be one and only one member of the Value List.	The entry must be one or multiple comma separated members of the Value List.<br><br>If OTH- is selected, then sequencing technology should be entered as free text.	The entry must include the forward primer name and nucleotide sequence separated by a colon.	The entry must include the reverse primer name and nucleotide sequence separated by a colon.	The entry must be the name of a valid sequence assembly program.	The entry must be the version number of the assembly program used in format v.x.x or the date the assemblies were created.	The entry must be a number or enter U if unknown.
**Examples**	25463985	viral cRNA	IonTorrent	fwd_seq:catgtttgcacaaagggaaa or U	rev_seq:atgtggatgcagattgtgga or U	IonTorrent Assembler	v.3.2 or 03-Mar-2011 or 3-Mar-2011 or 3-Mar-11	25.47
**Notes**	If the sequence was referenced in multiple publications, comma-separate the individual PMIDs.<br><br>Enter NA if the sequence was not referenced in a publication.	Enter viral cRNA for influenza virus sequences.<br><br>Enter genomic DNA for plasmid sequences or sequences of ribosomal RNA genes.	If more than one sequencing technology is used, comma-separate individual technologies.	If multiple forward primers were used, comma-separate individual forward primers.<br><br>Enter U if the forward primer is unknown.	If multiple reverse primers were used, comma-separate individual reverse primers.<br><br>Enter U if the reverse primer is unknown.	Sequences must be pre-assembled. Raw sequence reads from next generation sequencing technologies should not be submitted to GenBank.<br><br>If more than one assembly method is used, comma-separate individual methods.	If more than one assembly method is used, comma-separate individual versions.	If more than one coverage is used, comma-separate individual coverages.

	Genbank_Accession_Numbers	Strain_Name	Sample_Material	Surveillance_Sample	Host_Species	Host_Common_Name	Collection_Date	Collection_Country
**Input Type**	Text Field	Text Field	Text Field	Text Field	Text Field	Text Field	Date Field	Text Field
**Definition**	The GenBank accession number(s) for the sequence submission, if a GenBank number has been assigned	Name of the virus strain	Material on which the testing was performed. If multiple samples are taken from the same host, they must be entered as separate records.	Is the sequence derived from a surveillance sample?	Full scientific name of host genus and species, without abbreviations	The English common name given to a particular species	Date on which the sample was collected.	Country in which the original sample was collected
**Format**	Text<br><br>Maximum length: 150 characters	Antigenic Type/Host of Origin/Geographical Origin/Strain Number/Year of Isolation (Subtype)<br><br>rg-Antigenic Type/Host of Origin/Geographical Origin/Strain Number/Year of Isolation [rg details] (Subtype)<br><br>Maximum length: 100 characters	Text<br><br>Maximum length: 30 characters	Text<br><br>Maximum length: 1 character	Text<br><br>Maximum length: 50 characters	Text<br><br>Maximum length: 50 characters	DD-Mon-YYYY<br>DD-Mon-YY<br>Mon-YYYY<br>Mon-YY<br>YYYY<br>YY<br><br>Maximum length: 11 characters	Text<br><br>Maximum length: 60 characters
**Value List**	Text<br>NA	Text<br>U	AIR<br>BAL<br>BLO<br>CCF<br>CCO<br>CLO<br>FEC<br>LLF<br>LUN<br>NAL<br>NAS<br>NTS<br>ORP<br>OTH-<br>OTT<br>PLS<br>RCS<br>SER<br>SLU<br>SOI<br>SPU<br>TFB<br>TFT<br>TRS<br>WAT<br>U	Y<br>N	DPCC Species Dictionary<br>ENV<br>U	DPCC Species Dictionary<br>U	Date<br>U	ISO 3166 standard of country names
**Curation**	The entry must be a valid GenBank accession number or NA if none is available.	WHO strain naming convention: For non-reverse genetic viruses, fields must be ordered as follows and separated with the "/" character:<br>1. The antigenic type (e.g., A, B, C)<br>2. The host of origin (e.g., swine, equine, chicken. For human-origin viruses, no host of origin designation is given.)<br>3. Geographical origin (e.g., Denver, Taiwan)<br>4. Strain number (e.g., 15, 7)<br>5. Year of isolation (e.g., 2009, 1934)<br>6. For influenza A viruses, the hemagglutinin and neuraminidase antigen description in parentheses (e.g., (H1N1), (H3N2))<br><br>If the strain is a reverse genetic virus, prefix Strain_Name with rg-. Provide reverse genetic details in brackets between year of isolation and subtype.	The entry must be one and only one member of the Value List.<br><br>Values are case-sensitive and must be entered in all-caps.	The entry must be one and only one member of the value list.	If the entry is not ENV or U, the host species name is validated against the DPCC Species Dictionary.	The entry must be a text string or U.<br><br>If the entry is not U, the host common name is validated against the DPCC Species Dictionary.	1. Leading 0 in DD is optional.<br>2. Month must match the first three letters of the month. Month is NOT case-sensitive.<br>3. Years may have two or four digits.<br>4. Use U (Unknown) if date is not known.	The entry must be one and only one member of the Value List.<br><br>The DPCC will accept either the country name or (preferably) its unique ISO ALPHA-3 code in all-caps.
**Examples**	U12345 or AF123456 or NA	A/Hong Kong/1/1968 (H3N2),<br>A/swine/Iowa/233-56/2011 (H3N2v),<br>A/duck/Alberta//1976 (H1N1)<br>B/Hong Kong/432/2014,<br>C/Texas/19876/2011,<br>rg-A/Egypt/N03072/2010 [PR8 internal R 6+2] (H5N1),<br>rg-A/Puerto Rico/8/1934 (H1N1)	BLO	Y	Sus scrofa or Anser albifrons	Wild boar or White-fronted goose	3-Mar-2011 or 03-Mar-2011 or 03-MAR-2011 or Mar-2011 3-Mar-11 or 2011 or 11 or U	Viet Nam or VNM
**Notes**	If a GenBank_Accession_Number is provided, a FASTA file is not required.	Reference:<br>http://www.cdc.gov/flu/about/viruses/types.htm<br><br>(HxNx) can be used in cases where a partial subtype has been determined by viral isolation (e.g., H5Nx).<br><br>If there are mixed subtypes contained within a sample use A/chicken/Fujian/4/2002 (mixed) for Strain_Name or list multiple comma-separated Strain_Names: A/mallard/Alaska/2/2007 (H3N8), A/mallard/Alaska/2/2007 (H3N9).<br><br>For other virus types, enter the common Strain_Name.	AIR = Air<br>BAL = Bronchoalveolar lavage<br>BLO = Blood<br>CCF = Combined cloacal and fecal<br>CCO = Combined cloacal and oral-pharyngeal<br>CLO = Cloacal<br>FEC = Feces<br>LLF = Lung lavage fluid<br>LUN = Lungs<br>NAL = Nasal lavage<br>NAS = Nasal swab<br>NTS = Combined nasal throat swab<br>ORP = Oral-pharyngeal<br>OTH- = Other; append free text to describe<br>OTT = Other tissue<br>PLS = Plasma<br>RCS = Rectal swab<br>SER = Serum<br>SLU = Slurry<br>SOI = Soil<br>SPU = Sputum<br>TFB = Tissue from brain<br>TFT = Tissue from trachea<br>TRS = Tracheal swab<br>WAT = Water<br>U = Unknown	Y = Yes<br>N = No<br><br>Enter N if the sequence is a lab strain or reverse genetic virus.	Please reference the DPCC Species Dictionary for allowed values.<br><br>Use ENV(ironment) for samples taken from the environment (e.g., a water sample or feces picked up from the beach) when the source species is not known.<br><br>If genus is known but species is unknown, then use the genus followed by "sp" (for example domestic duck would be reported as "Anas sp").<br><br>If both the genus and species are unknown, then use the scientific family name, without abbreviation.<br><br>Enter U for all other cases.	Please reference the DPCC Species Dictionary for allowed values.<br><br>Use U if host common name is unknown or if the value entered under Host_Species is ENV or U.		The ISO 3166 standard of country names may be found on the DPCC Portal.

	Lab_Host	Parent_Strain_Name	Passage_History	Antigenic_Characterization	Treatment	Transmission_Method	Severity	Phenotype	Comments
Input Type	Text Field	Text Field	Text Field	Text Field	Text Field	Text Field	Text Field	Text Field	Text Field
Definition	Description of the lab host used for passaging the virus	Name of the parental virus strain.	Description of the passage history of the virus	Any information about antigenic characterization	Description of any experimental treatments	Description of the experimental method for virus transmission	Description of the severity of infection	Description of the viral phenotype	Text describing anything else of interest related to the submission
Format	Text<br><br>Maximum length: 50 characters	Antigenic Type/Host of Origin/Geographical Origin/Strain Number/Year of Isolation (Subtype)<br><br>Maximum length: 800 characters	Text<br><br>Maximum length: 50 characters	Text<br><br>Maximum length: 50 characters	Text<br><br>Maximum length: 50 characters	Text<br><br>Maximum length: 50 characters	Text<br><br>Maximum length: 50 characters	Text<br><br>Maximum length: 50 characters	Text<br><br>Maximum length: 200 characters
Value List	None	Text<br>U	None	None	None	None	None	None	Text<br>NA
Curation	None	WHO strain naming convention: Fields must be ordered as follows and separated with the "/" character:<br>1. The antigenic type (e.g., A, B, C, D)<br>2. The host of origin (e.g., swine, equine, chicken. For human-origin viruses, no host of origin designation is given.)<br>3. Geographical origin (e.g., Denver, Taiwan)<br>4. Strain number (e.g., 15, 7)<br>5. Year of isolation (e.g., 2009, 1934)<br>6. For influenza A viruses, the hemagglutinin and neuraminidase antigen description in parentheses (e.g., (H1N1), (H3N2))<br><br>If the strain is a reverse genetic virus, prefix Strain_Name with rg-.	None	None	None	None	None	None	None
Examples	Embryonated chicken egg	A/chicken/Fujian/4/2002 (H3N6), A/chicken/Fujian/4/2002 (HxNx), A/swine/Iowa/233-56/2011 (H3N2v), A/chicken/Fujian/4/2002 (mixed), B/Hong Kong/432/2014, or C/Texas/19876/2011	E2	NA	NA	Aerosol contact	NA	LPAI	NA
Notes		Reference: http://www.cdc.gov/flu/about/viruses/types.htm<br><br>(HxNx) can be used in cases where a partial subtype has been determined by viral isolation (e.g., H5Nx).<br><br>If there are mixed subtypes contained within a sample use A/chicken/Fujian/4/2002 (mixed) for Strain_Name or list multiple comma-separated Strain_Names: A/mallard/Alaska/2/2007 (H3N8), A/mallard/Alaska/2/2007 (H3N9).<br><br>For other virus types, enter the common Strain_Name.	Indicate lab host and number of passages:<br>E = Embryonated chicken eggs<br>C = MDCK cells<br>S = MDCK-SIAT cells<br>M = Monkey Kidney Cells<br><br>For other hosts, write out common name and include passage number e.g., Mouse2, E(duck)1, 293T1.<br><br>Use X if the host or passage number is unknown.<br><br>Enter NA if virus was not passaged.<br><br>Additional information relating to viral passaging can be entered in Comments.						If there are no comments, enter NA.
